# The Tumor Suppressor TGFBR3 Blocks Lymph Node Metastasis in Head and Neck Cancer

**DOI:** 10.3390/cancers12061375

**Published:** 2020-05-27

**Authors:** Wei-Yu Fang, Yi-Zih Kuo, Jang-Yang Chang, Jenn-Ren Hsiao, Hung-Ying Kao, Sen-Tien Tsai, Li-Wha Wu

**Affiliations:** 1 Institutes of Basic Medical Sciences, College of Medicine, National Cheng Kung University, Tainan 70101, Taiwan; tunafung@yahoo.com.tw; 2 Department of Otolaryngology, National Cheng Kung University Hospital, College of Medicine, National Cheng Kung University, Tainan 70101, Taiwan; u9360001@gmail.com (Y.-Z.K.); hsiaojr@mail.ncku.edu.tw (J.-R.H.); 3 National Institute of Cancer Research, National Health Research Institutes, Tainan 70456, Taiwan; jychang@nhri.org.tw; 4 Division of Hematology/Oncology, Department of Internal Medicine, National Cheng Kung University Hospital, College of Medicine, National Cheng Kung University, Tainan 70101, Taiwan; 5 Department of Biochemistry, School of Medicine, Case Western Reserve University, Cleveland, OH 43210, USA; hxk43@cwru.edu; 6 Institute of Molecular Medicine, College of Medicine, National Cheng Kung University, Tainan 70101, Taiwan; 7 Department of Laboratory Science and Technology, College of Health Sciences, Kaohsiung Medical University, Kaohsiung 80708, Taiwan

**Keywords:** TGFBR3, head and neck cancer, lymph node metastasis, GIPC1, ARRB2, angiogenin

## Abstract

The TGF-β type III receptor (TGFBR3) is an essential constituent of the TGF-β signaling. In this study, we observed a down-regulation of *TGFBR3* in oral cancer, a subtype of head and neck cancer (HNC), and patients with low *TGFBR3* had poor clinical outcomes. Ectopic expression of *TGFBR3* decreased migration and invasion of oral cancer cells and lymph node metastasis of tumors, whereas depletion of *TGFBR3* had the opposite effect. In SMAD4-positive OC-2 oral cancer cells, TGFBR3-mediated suppression requires both of its cytoplasmic interacting partners ARRB2 and GIPC1. We demonstrated that TGFBR3 induces the abundance of secreted angiogenin (ANG), a known pro-angiogenic factor, and ANG is essential and sufficient to mediate TGFBR3-dependent inhibition of migration and invasion of oral cancer cells. Notably, in *SMAD4*-deficient CAL-27 oral cancer cells, only GIPC1 is essential for *TGFBR3*-induced suppressive activity. Accordingly, HNC patients with low expressions of both *TGFBR3* and *GIPC1* had the poorest overall survival. In summary, we conclude that TGFBR3 is as a tumor suppressor via SMAD4-dependent and -independent manner in both tumor and stromal cells during oral carcinogenesis. Our study should facilitate the possibility of using TGFBR3-mediated tumor suppression for HNC treatment.

## 1. Introduction

Head and neck cancer (HNC) is a highly prevalent cancer worldwide [1]. Squamous cell carcinoma (SCC) constitutes 90% of oral cancer, a subtype of HNC. Despite current advances in surgery and treatment options, the survival rate of oral cancer patients has remained unchanged when compared to other cancer types [2]. Thus, a comprehensive understanding of molecular mechanisms underlying tumorigenesis and metastasis of HNC is vital for developing treatments for this cancer type.

Transforming growth factor-beta (TGF-β) pathway regulates cellular homeostasis, proliferation, motility, and angiogenesis in a cell type- and context-specific manner [3,4]. Among three surface receptors for TGF-β, type III receptor (TGFBR3), also known as betaglycan, is essential and the most abundant receptor that mediates TGF-β signaling [5,6]. As a high-affinity co-receptor for TGF-β, upon ligand binding, TGFBR3 facilitates ligand binding of TGFBR2, which phosphorylates and activates TGFBR1 kinase activity [5]. Activated TGFBR1 context-dependently induces SMAD-dependent canonical or non-canonical pathways by phosphorylation [4,7]. In the canonical pathway, TGFBR1-phosphorylated SMAD2/3 recruit SMAD4 to form a trimeric complex and translocate to the nucleus to regulate the expression of multiple target genes bearing SMAD binding elements [7,8]. TGFBR3 is a transmembrane protein with a short cytoplasmic domain, which interacts with β-arrestin 2 (ARRB2), as well as the adaptor protein, Gα-interacting protein-interacting protein C-terminus 1 (GIPC1) [9]. The phosphorylation of TGFBR3 by TGFBR2 is required for ARRB2 binding and subsequently its internalization, leading to the attenuation of TGF-β signaling [10,11]. GIPC1 binds the last three amino acids in the C-terminal PDZ-binding domain of TGFBR3 and modulates surface presentation of TGFBR3 [12]. Depending on the cellular context, TGFBR3 suppressed or facilitated cell migration and invasion via its interaction with ARRB2 or GIPC1 [13,14,15].

TGFBR3 has been shown to be a tumor suppressor in several cancer types, including oral cancer [16,17,18,19]. *TGFBR3* expression is decreased during tumor progression, and this decrease is associated with poor prognosis [16,17,18,19]. Ectopic expression of *TGFBR3* inhibits proliferation, migration, and invasion of cultured cells, and attenuated angiogenesis and metastasis in animal models. In contrast to its tumor-suppressive role, *TGFBR3* promotes oncogenesis in colon cancer and triple-negative breast cancer [20,21]. Increases of *TGFBR3* enhanced ligand-stimulated anchorage-independent growth and migration of colon cancer cells while modestly increasing tumorigenesis of xenografted animals [20]. Consistently, depletion of *TGFBR3* blocked ligand-induced stimulatory effect on breast cancer cell motility, invasion, and xenograft tumor growth [21]. These results support the notion that TGFBR3 regulates cancer progression in a context-dependent manner. Nonetheless, the mechanistic insights into the role of TGFBR3 in HNC carcinogenesis remain unexplored.

The interactions between epithelial cells and their microenvironment, a heterogeneous mixture of stromal cells including cancer-associated fibroblasts (CAFs), endothelial cells, and immune cells, play a vital role in the initiation and development of cancer [22]. Among stromal cells, CAFs play a dominant role in the tumor microenvironment and contribute to an aggressive cancer phenotype via promoting proliferation, facilitating invasiveness, and suppressing host immune system [23]. TGF-β-mediated signaling in fibroblasts modulates the growth and oncogenic potential of adjacent epithelial cells in selected tissues [24]. In support of this notion, *TGFBR3* is downregulated in oral cancer-associated fibroblasts relative to that in normal fibroblasts (NFs) and negatively regulated by TGF-β in CAFs [19] and cancer cells [25]. However, the role and action mechanism of TGFBR3 in mediating the crosstalk between oral cancer cells and stromal cells remains elusive.

The present study aims to dissect the role of TGFBR3 in oral cancer cells and examine the molecular mechanism whereby TGFBR3 mediates the crosstalk between HNC and stromal cells.

## 2. Results

### 2.1. Decreased TGFBR3 Expression in Oral Cancer Clinical Specimens

**Figure 1:**
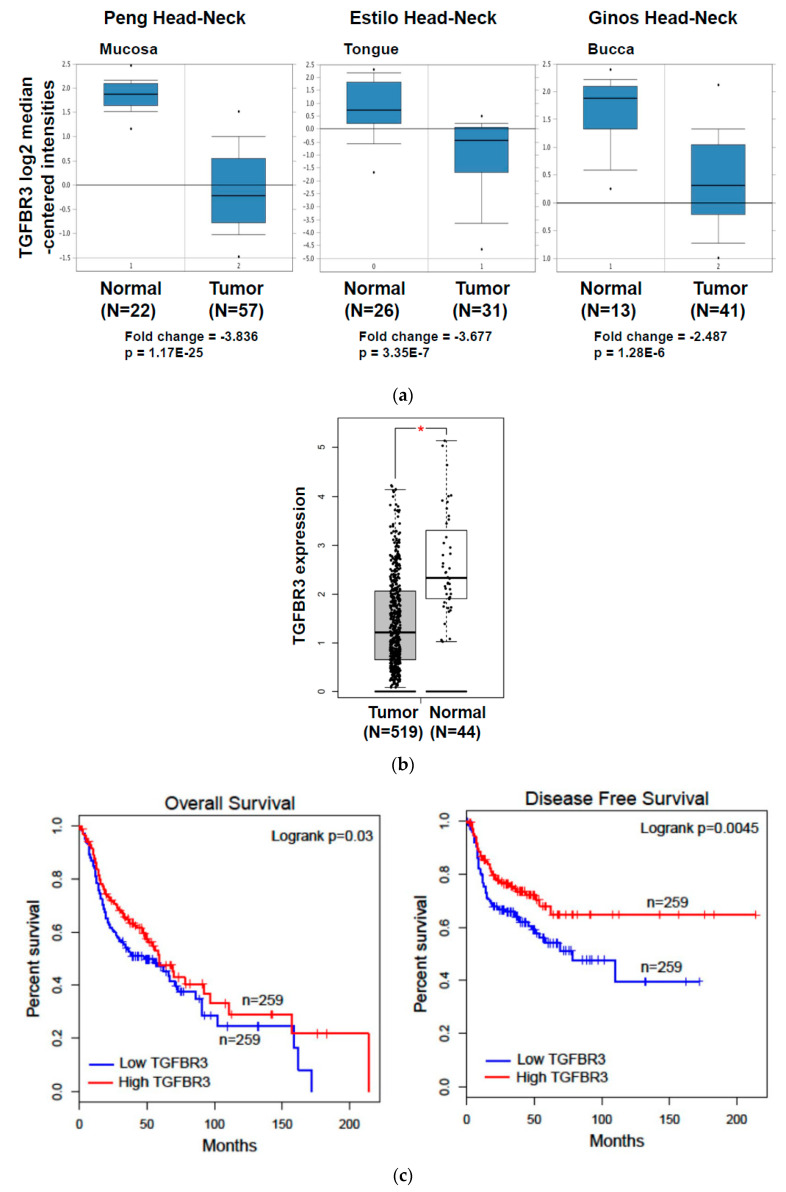

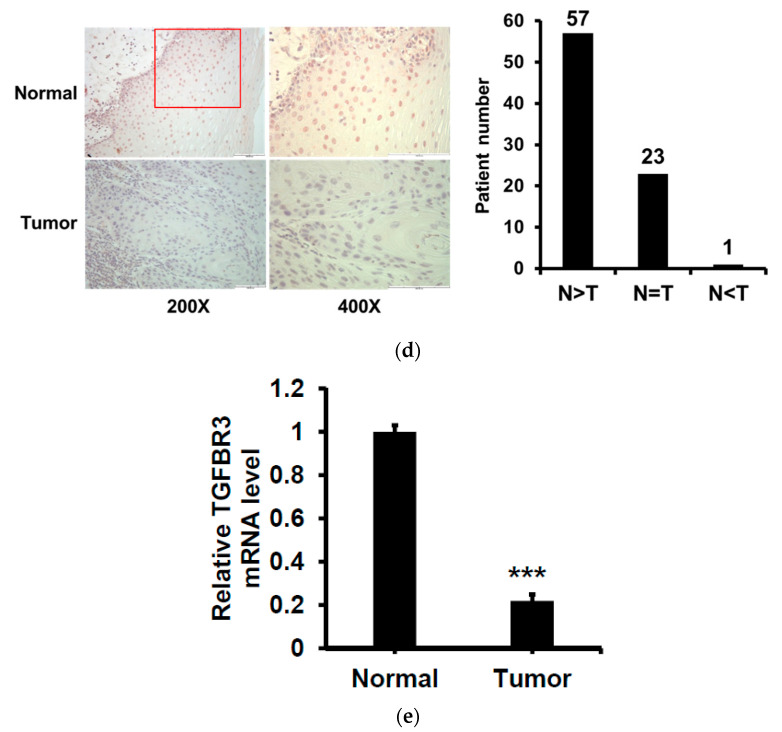
The expression of TGFBR3 mRNA in TCGA-HNC patient cohorts and oral cancer and its impacts on patient clinical outcomes. (**a**) Oncomine analysis of TGFBR3 mRNA expression in HNSCC. We used box-plot diagrams to compare the mRNA levels of TGFBR3 in normal tissues with those in tumor tissues using Oncomine datasets. (**b**) The expression of TGFBR3 mRNA in HNC tissues (*N* = 519), and normal tissues (*N* = 44) in TCGA, as accessible on the GEPIA website (* *p* < 0.05). (**c**) The overall survival and disease-free survival rates of HNC patients were analyzed using a log-rank test based on high (>median) and low (<median) TGFBR3 mRNA levels from the TCGA cohort. Kaplan–Meier curves were plotted for TGFBR3 using the GEPIA web server. (**d**) Left: Representative IHC images showed a decrease in TGFBR3 expression in tumor specimens from oral cancer (*N* = 81). Right: Patient numbers in three groups based on the staining intensity of TGFBR3 in tumors relative to paired normal tissues. (**e**). The TGFBR3 mRNA expression in tumor tissues compared to those in adjacent healthy tissues (*N* = 86) by RT-qPCR. Each experiment was performed in triplicates. Data are Mean ± SEM. *** *p* < 0.001 vs. normal.

To investigate whether *TGFBR3* expression was dysregulated in oral cancer specimens, we first performed an in silico analysis by using publicly available gene expression datasets for HNC from Oncomine and The Cancer Genome Atlas (TCGA). Oral cancer is a subtype of HNC. Through the analysis of three studies [26,27,28], the expression of *TGFBR3* mRNA was 3.836-fold lower in oral cancer tissues than that in the normal oral mucosa (*p* = 1.17 × 10^−25^), 3.677-fold lower in tongue cancer tissues than that in healthy tongues (*p* = 3.35 × 10^−7^), and 2.487-fold lower in HNC tissues than that in the normal buccal mucosa (*p* = 1.28 × 10^−6^) (Figure 1a). Further analysis of the TCGA data by Gene Expression Profiling Interactive Analysis (GEPIA) [29] showed a decrease in *TGFBR3* mRNA expression in HNC compared to healthy tissues (Figure 1b). This low *TGFBR3* mRNA expression was associated with poor overall and disease-free survival among these patients (Figure 1c), suggesting a critical role of *TGFBR3* in the pathogenesis of HNC.

To validate the above findings in *TGFBR3* mRNA expression, we examined the protein expression of TGFBR3 in 81 oral cancer specimens from NCKU by IHC staining. We found that 70% (57/81) of these specimens showed a decrease in TGFBR3 protein expression in tumor tissues compared to adjacent healthy tissues (Figure 1d and Figure S1). Similarly, we observed a significant reduction in *TGFBR3* mRNA expression in tumor tissues compared to the healthy tissues (Figure 1e). Collectively, these data indicated that *TGFBR3* mRNA and protein abundance were significantly lower in oral cancer and suggested a role of TGFBR3 in the oncogenesis of oral cancer.

### 2.2. The Effects of TGFBR3 on Proliferation, Migration, and Invasion of Oral Cancer Cells

**Figure 2:**
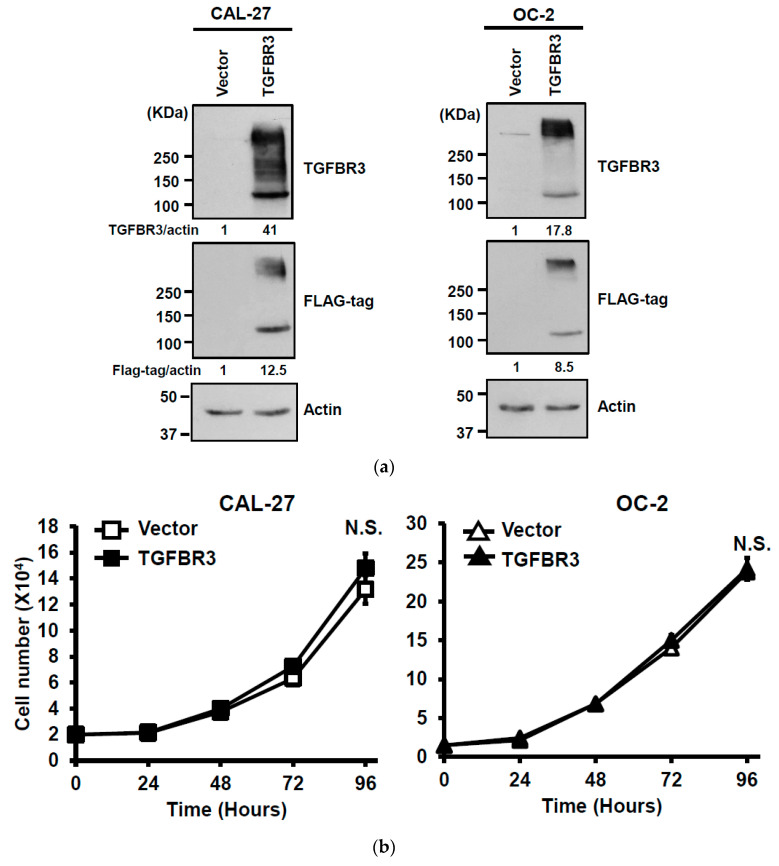

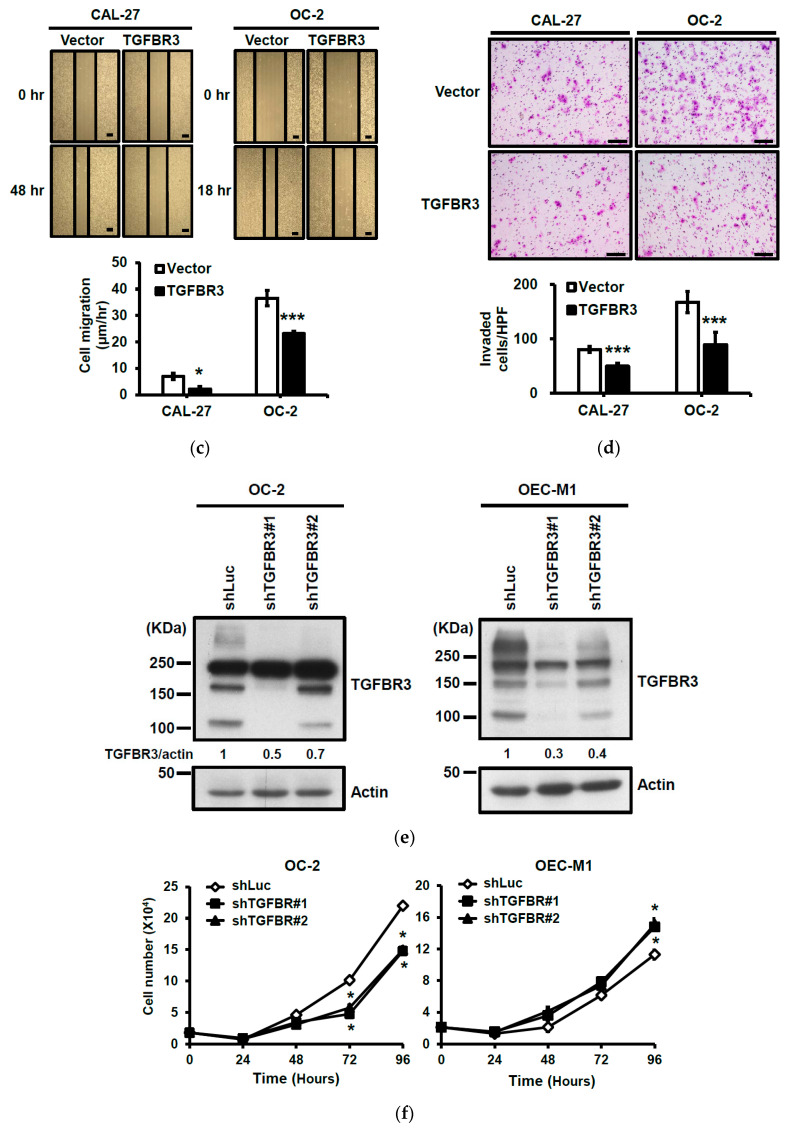

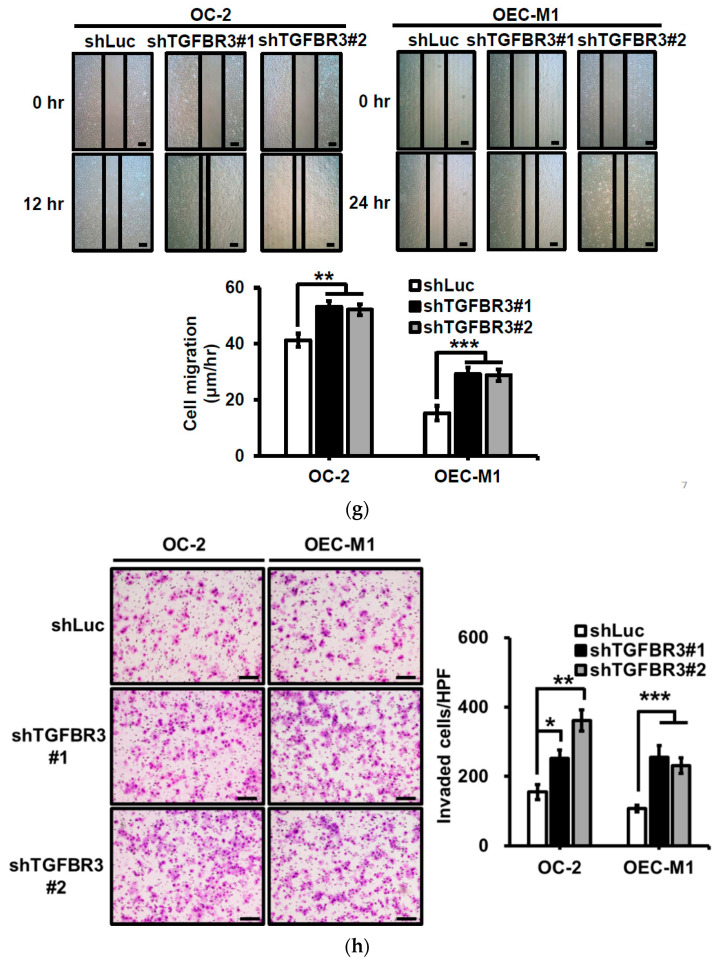
The effect of TGFBR3 on oral cancer cell behaviors. (**a**) CAL-27 or OC-2 cells were infected with lentiviruses bearing empty vector or FLAG-tagged TGFBR3 followed by Western blot analysis. The blot is a representative of three independent experiments. Actin was used as a loading control. These cells were further subjected to cell proliferation (**b**), wound repair (**c**), and Matrigel invasion, scale bar: 200 μm. (**d**) assays. Scale bar: 200 μm. (**e**) OC-2 or OEC-M1 were infected with lentiviruses bearing shLuc or sh*TGFBR3* (clone #1 or #2) to deplete *TGFBR3* expression in the cells. The blot is a representative of three independent experiments. Actin was used as a loading control. (**f**) The numbers of the viable transfected cells were enumerated by cell proliferation assay. Cell migration, scale bar: 200 μm (**g**) and invasion Scale bar 200 μm (**h**) were measured, respectively, by wound repair and Matrigel invasion assays. All data are representatives of two-three independent experiments, each performed in triplicates. * *p* < 0.05, ** *p* < 0.01, *** *p* < 0.001 or N.S. (not significant) versus control or in the indicated group. Student’s *t*-test. The uncropped blots with molecular weight markers for Figure 2a,e are, respectively, shown in Figures S9 and S10.

To dissect the role of TGFBR3 in oral cancer cells, we first examined the abundance of TGFBR3 in a panel of oral cancer lines (Figures S2a and S19). The OC-2 cells express wild-type SMAD4, whereas CAL-27 cells harbor a nonsense mutation in the *SMAD4* gene ([30], Figures S2b and S19). Thus, we engineered cell lines in which *TGFBR3* is stably overexpressed in these two cell lines using a lentiviral expression system. In addition to the presence of core TGFBR3 protein around 100 kDa, we also detected several protein bands migrating at a range of 150 to 300 kDa in these overexpressing cells (Figure 2a), possibly due to post-translational modifications [3,21]. Although little or no effect on the proliferation of CAL-27 or OC-2 oral cancer cell lines was observed (Figure 2b), overexpression of *TGFBR3* significantly decreased cell migration and invasion of both lines (Figure 2c,d). Furthermore, the silencing of endogenous *TGFBR3* in OC-2 or OEC-M1 cell lines (Figure 2e) increased migration (Figure 2g) and invasion (Figure 2h) while it differentially affected cell proliferation (Figure 2f).

### 2.3. Ectopic TGFBR3 Expression Decreased Lymph Node Metastasis without Affecting Xenograft Tumor Growth

**Figure 3:**
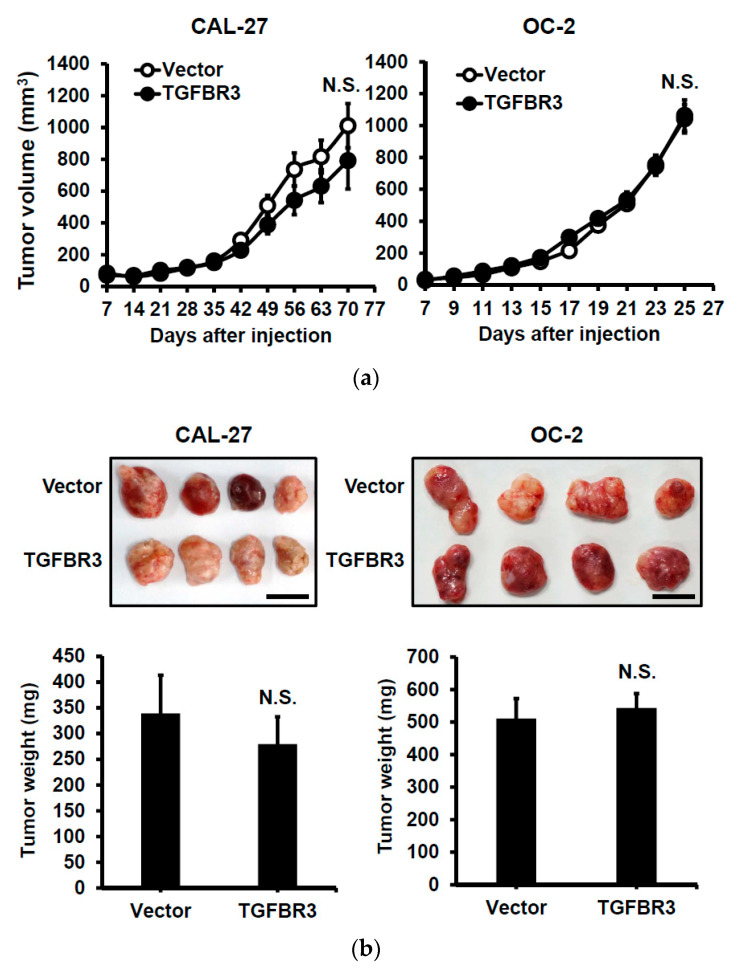

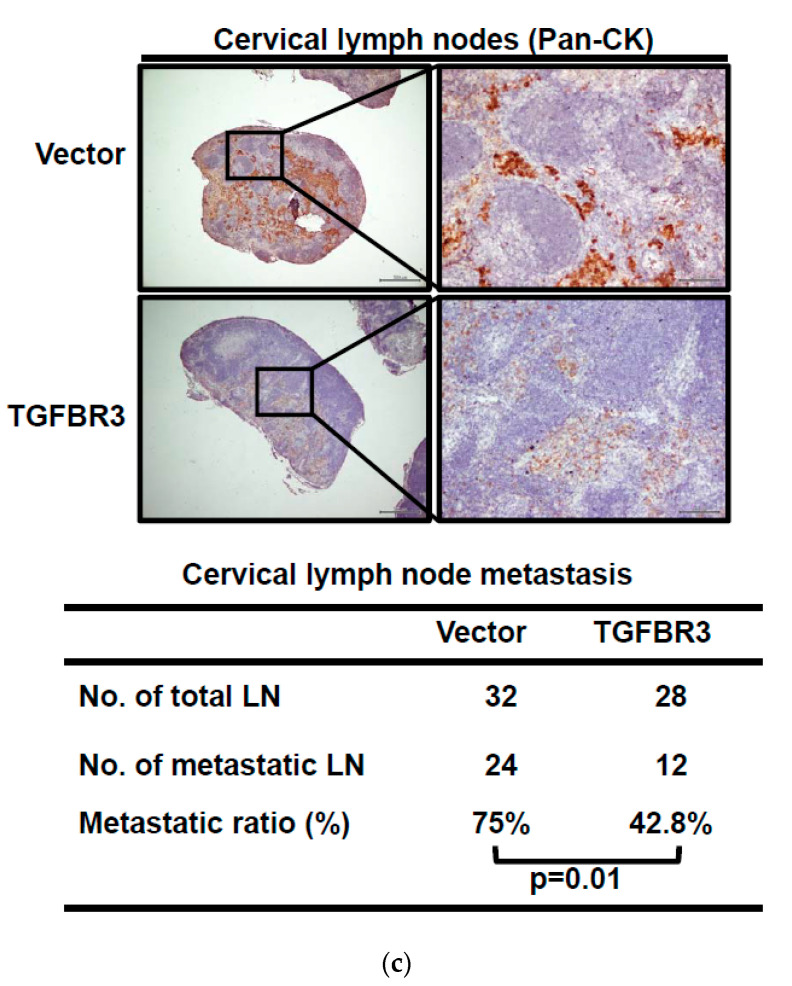
Ectopic *TGFBR3* expression decreased metastasis without affecting primary tumor growth. (**a**) TGFBR3- or vector-stably transfected CAL-27 cells (left) or OC-2 (right) were subcutaneously injected into male nude mice (8 mice per group). Tumor sizes were measured on the indicated days. (**b**) The images and tumor weights of vector or TGFBR3 group were taken at the endpoint. Scale bar, 1 cm. (**c**). TGFBR3-transfeced or control OC-2 cells were orthotopically injected into the buccal mucosa of male nude mice (7–8 mice per group) for 46 days. Top: IHC staining for pan-CK-positive tumor cells in cervical lymph nodes (×40 and ×200). Scale bar, 100 μm. Bottom: Metastatic ratios of cervical lymph nodes; the Chi-square test was used for statistical analysis. N.S., not significant versus vector control. Student’s *t*-test.

To investigate the effect of *TGFBR3* expression on xenograft tumorigenesis, we subcutaneously injected vector or *TGFBR3*-expressing cells onto male nude mice. Interestingly, neither the primary tumor growth nor the final tumor weight were affected by *TGFBR3* overexpression (Figure 3a,b). To test whether *TGFBR3* overexpression affects tumor metastasis, we injected the mice intrabuccally with vector- or TGFBR3-expressing OC-2 cells. The cervical lymph nodes were collected at endpoints and immunostained with pan-cytokeratin (Pan-CK) antibodies to detect tumor cell-positive lymph nodes. We found that overexpression of *TGFBR3* decreased metastasis of cervical lymph nodes (Figure 3c).

### 2.4. TGFBR3 Inhibits TGF-β1-Mediated Signal Transduction, Migration, and Invasion in Oral Cancer Cells

**Figure 4:**
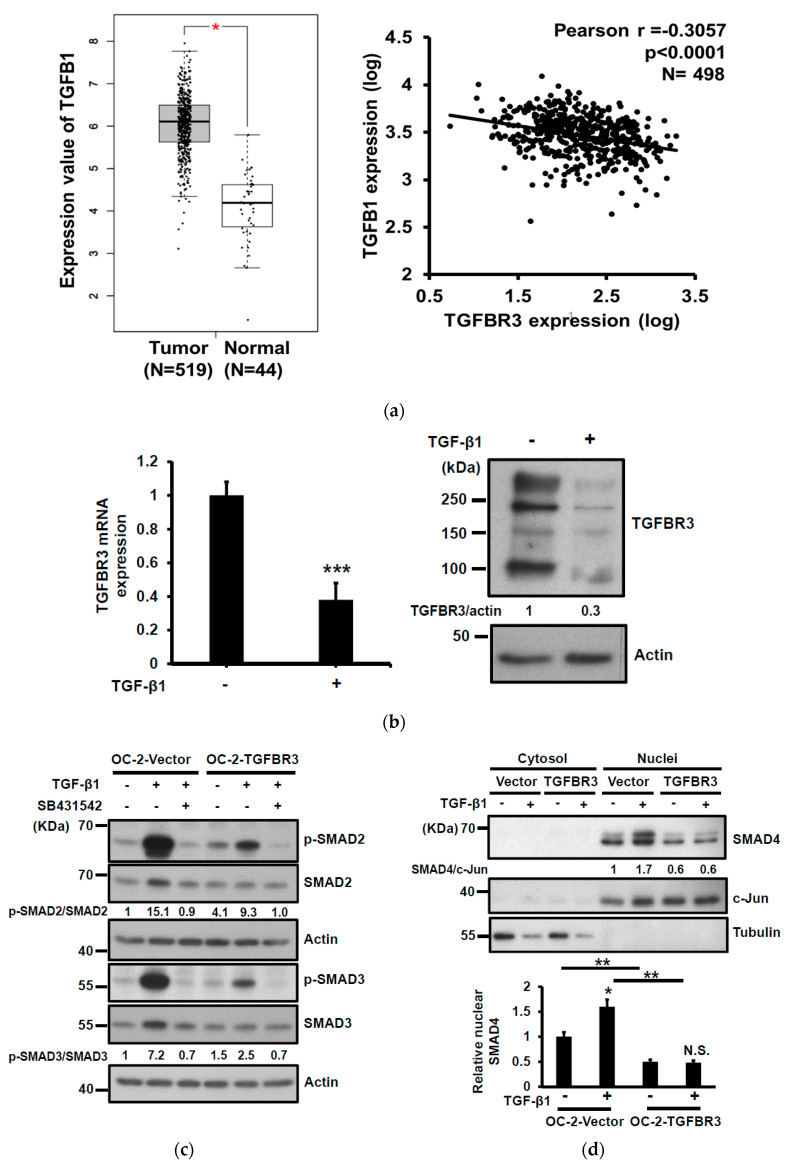

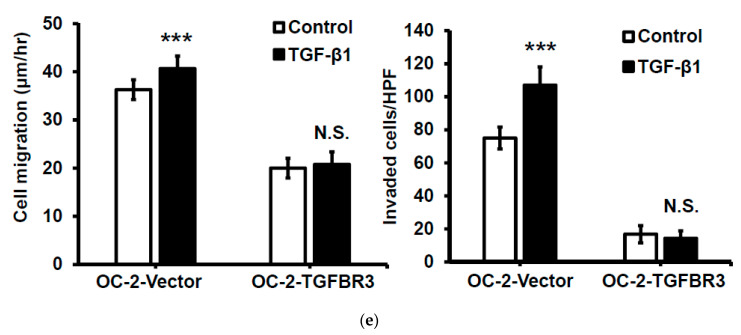
TGFBR3 inhibited TGF-β1-mediated signal transduction, cell migration, and invasion in oral cancer cells. (**a**) Left: *TGFB1* mRNA expression in HNC tissues (*N* = 519), and normal tissues (*N* = 44) was analyzed based on the TCGA dataset, as accessible on the GEPIA website (* *p* < 0.05). Right: Pearson correlation analysis showed a negative correlation of *TGFB1* and *TGFBR3* mRNA expression in tumor specimens from the TCGA-HNC dataset following log-transformation. (**b**) The effect of TGF-β1 on the mRNA (left) and protein (right) levels of TGFBR3 in the OEC-M1 cells. (**c**) The effect of TGFBR3 overexpression on total and phosphorylated SMAD2/SMAD3 in the absence or presence of TGF-β, with or without SB431542. Actin was used as a loading control. (**d**) The effect of overexpression of TGFBR3 on the nucleocytoplasmic distribution of SMAD4. Tubulin and c-Jun were used as loading controls for cytosol and nucleus, respectively. Bottom, the quantitative result of three independent repeats of nuclear SMAD4. ** *p* < 0.01. (**e**). The effect of overexpression of TGFBR3 on migration (left) and invasion (right) of OC-2 cells in the presence of TGF-β. The data were expressed as mean ± SD. *** *p* < 0.001 or N.S. (not significant) versus vector; Student’s *t*-test. The uncropped blots with molecular weight markers for Figure 4b–d are individually shown in Figures S11–S13.

To elucidate the potential mechanism involved in the down-regulation of *TGFBR3* in clinical specimens, we first used cBioPortal [31] to study the genetic status of the *TGFBR3* gene. Only 1.2% of genetic alterations in *TGFBR3* were identified in 504 HNC patients (Figure S3), which is unlikely to account for the majority of the clinical specimens exhibiting low *TGFBR3* expression (Figure 1). It was previously shown that TGF-β1 negatively regulates *TGFBR3* mRNA expression through the inhibition of its proximal promoter [25]. We analyzed the correlation between *TGFB1* and *TGFBR3* expression in the TCGA-HNC database. We found an increase of *TGFB1* mRNA in tumor tissues relative to healthy ones (Figure 4a, left), and an increase of *TGFB1* mRNA was inversely correlated with a decrease of *TGFBR3* mRNA levels in the same patients (Figure 4a, right). TGF-β1 stimulation readily reduced both mRNA and protein expression of TGFBR3 in high TGFBR3-expressing OEC-M1 cells (Figure 4b).

TGFBR3 is a co-receptor that stimulates or inhibits the canonical TGF-β signaling pathway [5,32]. To elucidate the role of TGFBR3 in TGF-β signaling in oral cancer cells, we determined the effect of *TGFBR3* expression on phosphorylation of SMAD2/3, an activation mark of the canonical TGF- β pathway, with or without SB431542, an inhibitor of TGFBR1. As expected, SB431542 blocked SMAD2/3 phosphorylation in both vector- and TGFBR3-transfected cells (Figure 4c). Notably, although overexpression of *TGFBR3* had little effect on basal SMAD2/3 phosphorylation, it attenuated TGF-β1-stimulated SMAD2/3 phosphorylation. Nucleocytoplasmic fractionation, followed by Western blot analysis, also showed that the abundance of nuclear SMAD4 was significantly reduced in TGFBR3-expressing cells (Figure 4d). Because TGF-β1 enhanced migration and invasion of HNC cells [33], we next investigated the role of TGFBR3 in TGF-β1-induced migration and invasion of oral cancer cells. We found that *TGFBR3* overexpression abolished TGF-β1-induced migration and invasion of OC-2 HNC cells (Figure 4e). Overall, these data indicated that *TGFBR3* and TGF-β1 antagonize each other.

### 2.5. A Requirement of the Scaffolding Protein GIPC1 for TGFBR3-Mediated Inhibition of Migration and Invasion of SMAD4-Deficient Oral Cancer Cell

**Figure 5:**
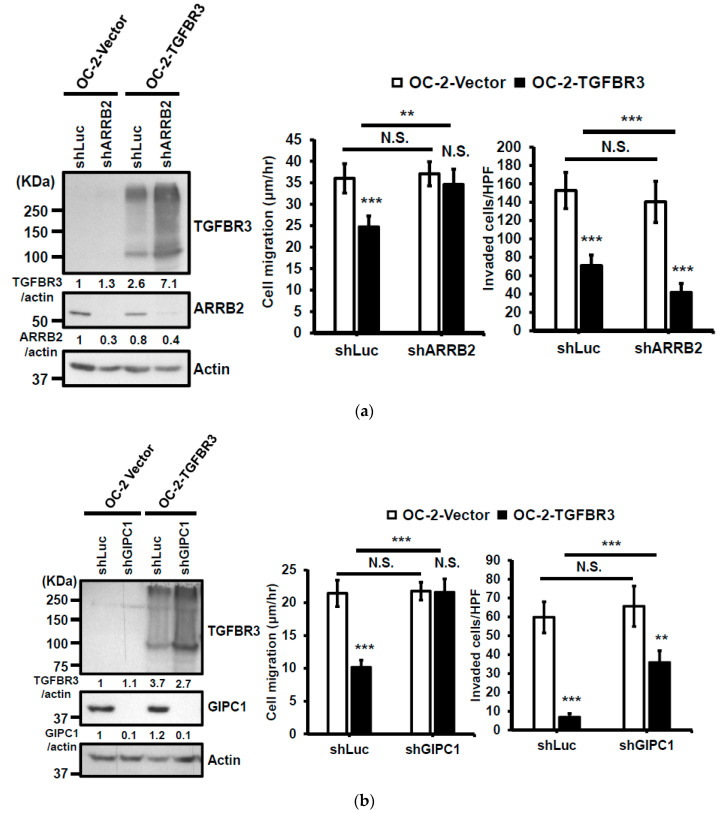

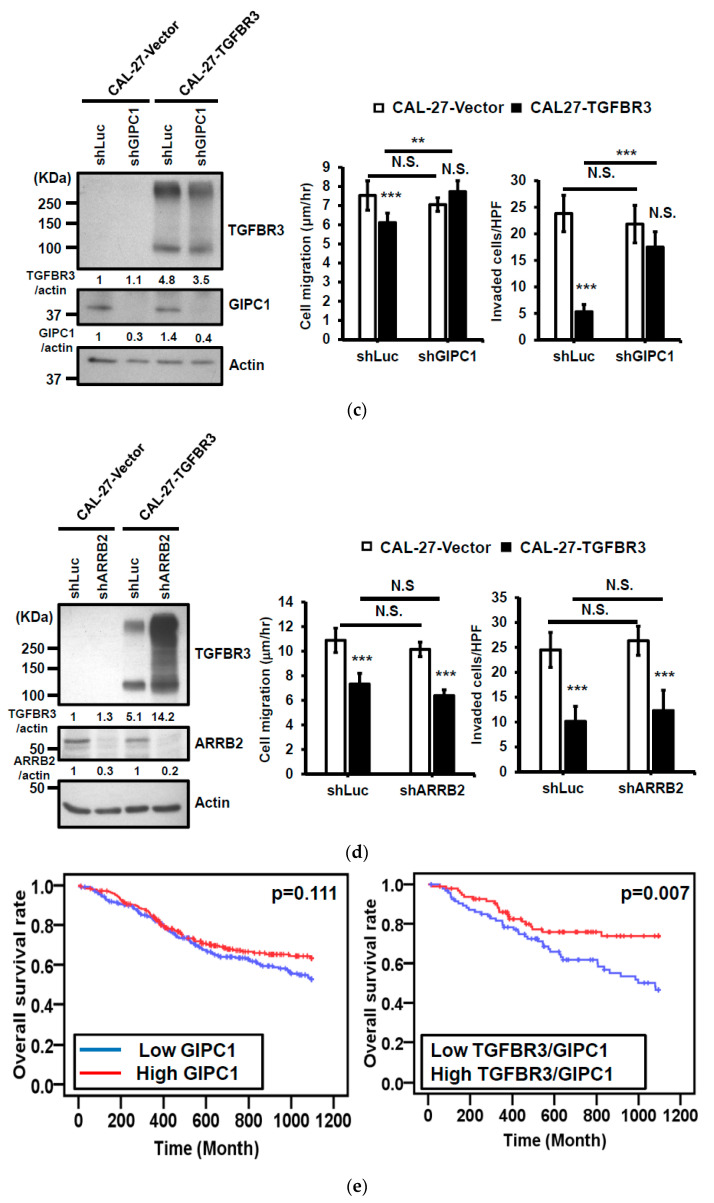
GIPC1 scaffolding protein functioned in the same signaling axis as TGBFR3-mediated suppression. (**a**,**b**) Left: Western blot analysis of ARRB2 (**a**) or GIPC1 (**b**) and TGFBR3 protein levels. Actin was used as a loading control. Right: The effects of sh*GIPIC1* (**a**) or sh*ARRB2* (**b**) on TGFBR3-dependent suppressive effects on OC-2 cells. Data are presented as mean ± SD. (**c**,**d**) Similar experiments were performed as a–b, except that CAL-27 cells were analyzed. (**e**) Kaplan–Meier analysis shows a correlation of overall survival with the expression of GIPC1 (left) and GIPC1/TGFBR3 (right) in the TCGA-HNC dataset. The concordant decrease of both TGFBR3 and GIPC1 expression in these patients had reduced overall survival relative to those with both high expressions (*p* = 0.007). ** *p* < 0.01, *** *p* < 0.001 or N.S. (not significant) versus vector; Student’s *t*-test. All the uncropped blots with molecular weight markers for Figure 5a–d are shown in Figures S14–S17.

The cytosolic domain of TGFBR3 mediates TGF-β signaling by interacting with ARRB2 and GIPC1 [13,14,15]. We sought to dissect whether ARRB2 or GIPC1 plays a role in TGFBR3-dependent cancer-suppressive activity. In OC-2 cells, knockdown of *ARRB2* mitigated TGFBR3-induced inhibition of cell migration without affecting cell invasion (Figure 5a). In contrast, in *GIPC1* knockdown OC-2 cells, both TGFBR3-induced reduction of cell migration and invasion were significantly blocked (Figure 5b). Overexpression of *TGFBR3* also significantly inhibited migration and invasion of CAL-27 cells, implying both SMAD4-dependent and independent pathways (Figure 2c–d). We next investigated whether ARRB2 or GIPC1 plays a role in SMAD4-independent, TGFBR3-mediated migration and invasion of oral cancer cells by knocking down *ARRB2* or *GIPC1* in *TGFBR3*-overexpressing CAL-27 cells. We found that only the silencing of *GIPC1* (Figure 5c) but not *ARRB2* (Figure 5d) significantly blocked TGFBR3-mediated suppression of cell migration and invasion (Figure 5b–c). These data suggest a critical role of GIPC1 in TGFBR3-mediated inhibition of migration and invasion in both OC-2 and CAL-27 oral cancer cells. Consistently, the survival curve analysis indicated that although low *GIPC1* expression alone was marginally associated with reduced overall survival in TCGA-HNC (*p* = 0.111) (Figure 5e, left), a concordant reduction of *GIPC1* and *TGFBR3* expression significantly reduced patient clinical outcomes (*p* = 0.007) (Figure 5e, right). No such correlation between TGFBR3 and ARRB2 was detected in the same patient groups (Figure S4).

### 2.6. Suppressive Effect of Conditional Medium (CM) from TGFBR3-Expressing Cancer Cells on Cancer Cells, Fibroblasts, and Endothelial Cells (ECs)

**Figure 6:**
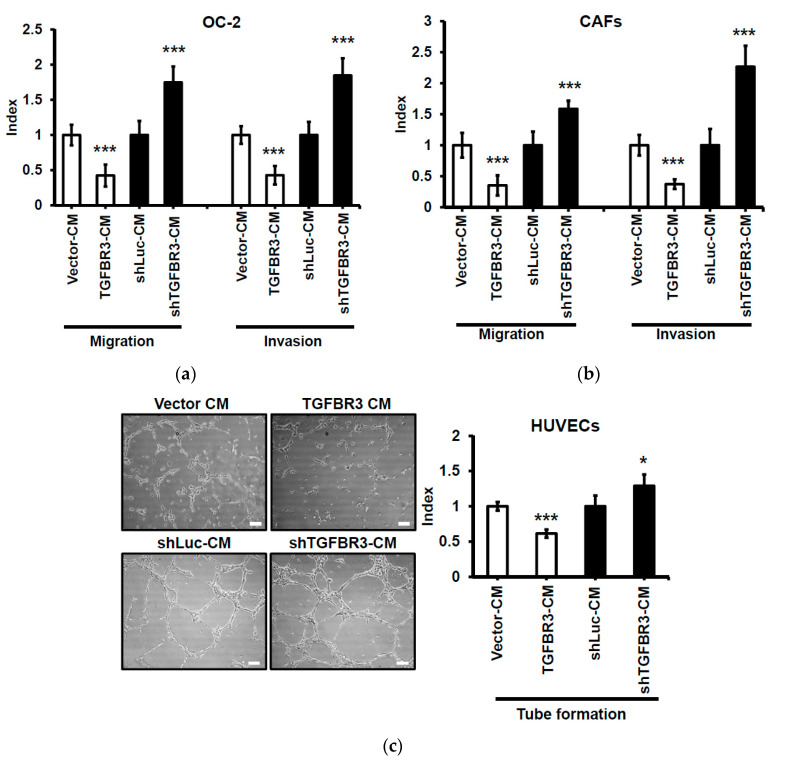
Suppressive effect of CM from *TGFBR3*-overexpressing cancer cells on oral cancer cells, CAFs, and endothelial cells. Following treatment of the indicated cells with CM from TGFBR3-manipulated OC-2 cells, cell migration (Left) and invasion abilities (Right) of the OC-2 cells (**a**) were respectively measured by wound repair and Matrigel invasion assays. (**b**). The migration (Left) and invasion (Right) abilities of CAFs were, respectively, measured by wound repair and collagen invasion assays. (**c**) Endothelial formation assay of HUVECs following the treatment with CM from TGFBR3-manipulated OC-2 cells, scale bar: 100 μm. Endothelial vessel numbers were measured. Data are expressed as mean ± SD. * *p* < 0.05, ** *p* < 0.01, *** *p* < 0.001 or N.S. (not significant) versus shLuc-CM; Student’s *t*-test.

TGF-β is a pleiotropic growth factor affecting both tumor and stromal cells in tumorigenesis [34]. We further examined the paracrine effect of CM derived from *TGFBR3*-manipulated oral cancer cells on cancer cells, CAFs, and ECs. We observed that CM collected from *TGFBR3*-overexpressing OC-2 cells decreased migration and invasion of OC-2 cells (Figure 6a) and CAFs isolated from oral cancer specimens (Figures S5 and S20 and Figure 6b), and inhibited tube formation of human umbilical vein ECs (HUVECs) (Figure 6c), without affecting proliferation of CAFs or ECs (Figure S6). Conversely, CM collected from *TGFBR3* knockdown cells had the opposite effects on stromal cells (Figure 6a–c).

### 2.7. Angiogenin (ANG) Is a Novel TGFBR3 Downstream Target that Mediates Its Tumor Suppression Activity

**Figure 7:**
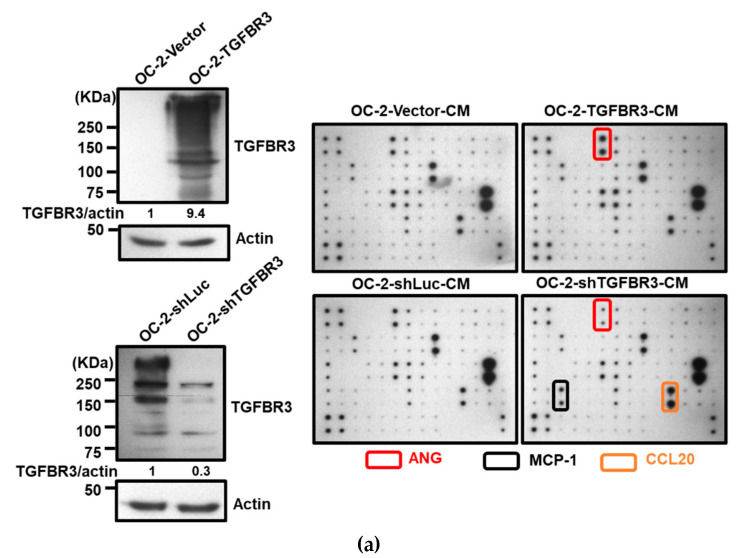

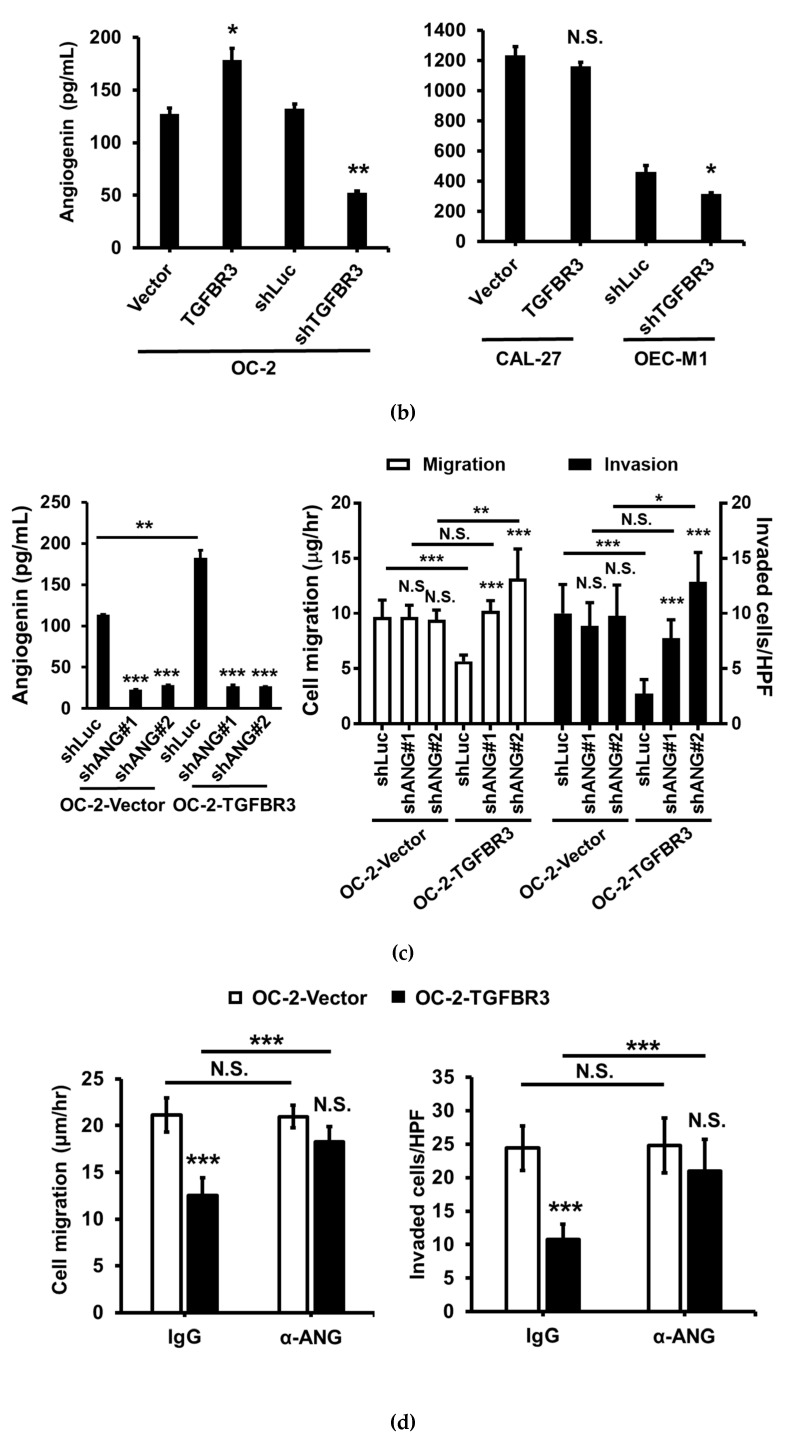

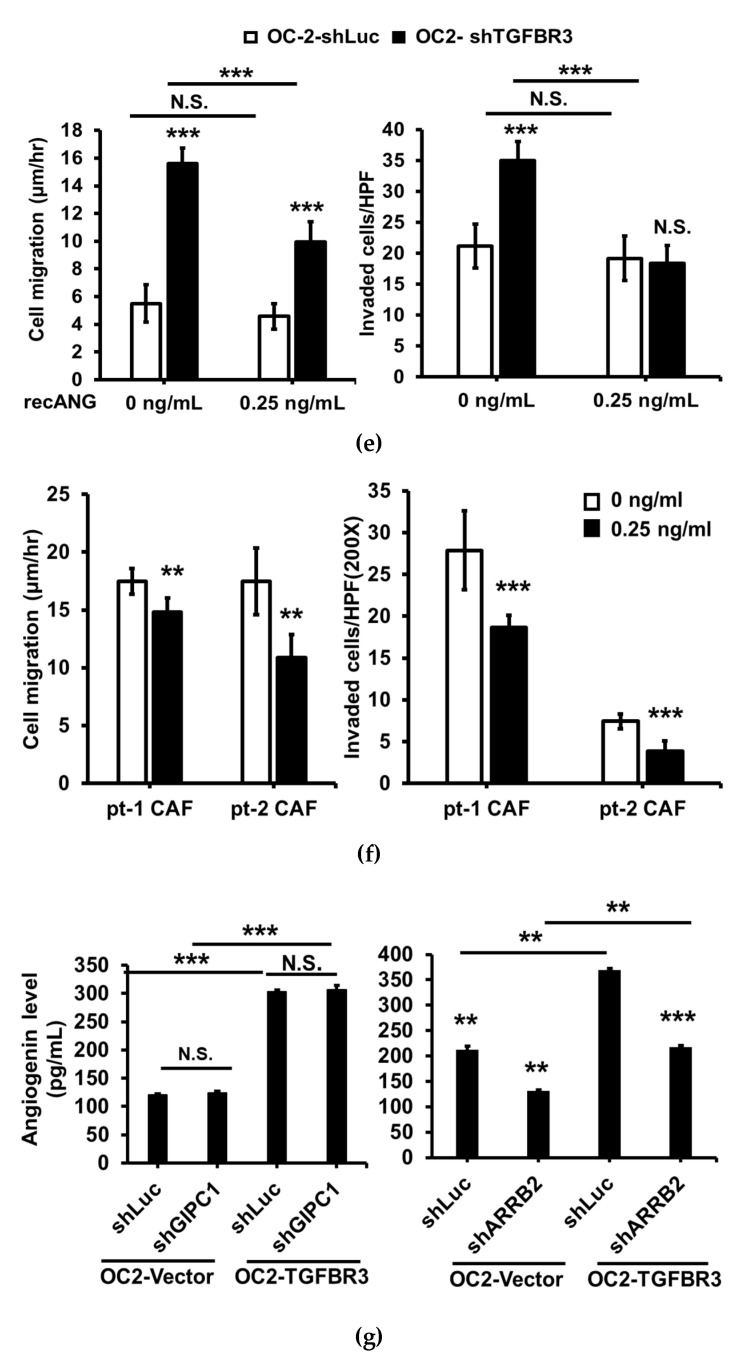
ANG is a novel downstream effector of TGFBR3. (**a**). Left: Western blotting of TGFBR3 in overexpression and knockdown OC-2 cells. Right: The CM obtained from TGFBR3 overexpression (top) or knockdown (bottom) OC-2 cells were used to analyze differential expression secreted cytokines by using C-Series Human Cytokine Antibody Array 1000 Kits. The differential intensities of ANG, MCP-1, and CCL20 protein in the indicated CM were boxed with the indicated colors. (**b**) The effects of TGFBR3 on the abundance of secreted ANG protein in OC-2 cells (left), CAL-27 and OEC-M1 (right). (**c**) The effect of silencing *ANG* on the abundance of TGFBR3-induced secreted ANG (left) and inhibition of OC-2 cell migration and invasion (right). (**d**) The effect of anti-ANG antibodies on TGFBR3-induced inhibition of migration (left) and invasion (right) of OC-2 cells. (**e**) The effects of recombinant ANG (recANG) on migration (left) and invasion (right) of *TGFBR3*-knockdown OC-2 cells. (**f**) The effect of recANG on the migration (left) and invasion (right) of CAFs isolated from two oral cancer patients. (**g**) The effect of knockdown of *GIPC1* (left) and *ARRB2* (right) on TGFBR3-dependent increases in ANG levels in OC-2 cells. Data are presented as mean ± SD. * *p* < 0.05, ** *p* < 0.01, *** *p* < 0.001 or N.S. (not significant) versus shLuc-CM; Student’s *t*-test. All the uncropped blots with molecular weight markers for Figure 7a are shown in Figure S18.

We hypothesized that secretary cytokines from TGFBR3-overexpressing OC-2 cells exert their effects on the stromal cells. To test this, we compared the secreted protein profiles of CM prepared from *TGFBR3*-overexpressing OC-2 cells (Figure 7a, top) to those collected from *TGFBR3*-depleted OC-2 cells (Figure 7a, bottom) by using a cytokine antibody array. We found that the alterations of *TGFBR3* expression significantly influenced the levels of three cytokines, MCP-1, CCL20, and angiogenin (ANG) (Figure 7a, right). However, only ANG was concordantly expressed with that of TGFBR3. ELISA was used to validate the concordant correlation between TGFBR3 and ANG in the CM derived from TGFBR3-manipulated OC-2 cells (Figure 7b, left) and OEC-M1 cells (Figure 7b, right). However, the effects of TGFBR3 on secreted ANG protein abundance was not observed in CAL-27 cells (Figure 7b, right). The ability of TGFBR3 to induce secreted ANG was further confirmed in SMAD4-positive 293T cells (Figures S7 and S21). We next tested whether TGFBR3-mediated tumor-suppressive function depends on ANG. We found that inhibition of ANG by shRNA knockdown in TGFBR3 stably-expressed OC-2 cells significantly decreased secreted ANG protein (Figure 7c, left) and abolished TGFBR3-dependent inhibition of OC-2 cell migration and invasion (Figure 7c, right). The addition of anti-ANG antibodies similarly blocked TGFBR3-dependent inhibition of migration and invasion of OC-2 cells (Figure 7d). Furthermore, while the addition of recombinant ANG (recANG) protein partially attenuated *TGFBR3* knockdown-induced cell migration, it completely blocked the invasion of *TGFBR3*-depleted OC-2 cells (Figure 7e). Exogenous ANG also suppressed CAF migration and invasion (Figure 7f). These data indicated that secreted ANG induced by TGFBR3 exerts its suppressive effects on the migration and invasion of OC-2 oral cancer cells and CAFs. We further dissected the role of ARRB2 and GIPC1 in TGFBR3-mediated secretion of ANG and found that only loss of *ARRB2*, not *GIPC1*, had significant effects on the secreted ANG abundance (Figure 7g).

## 3. Discussion

**Figure 8:**
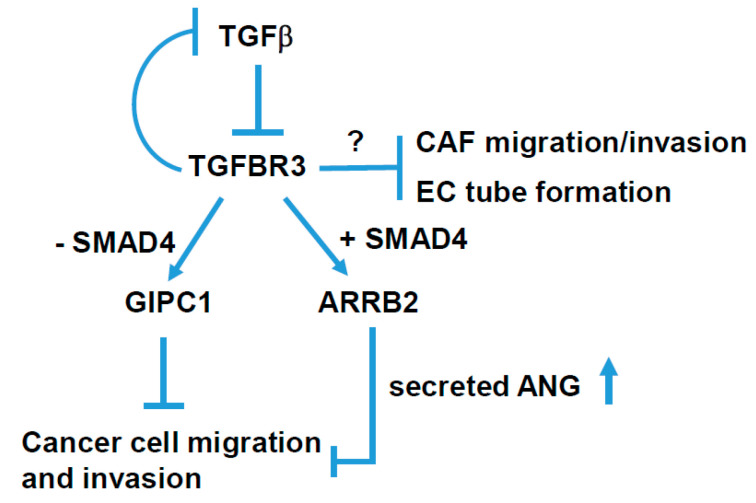
A schematic model for TGFBR3-mediated tumor-suppressive signaling in head and neck cancer. TGFBR3 exerts a tumor-suppressive effect in both tumor and stroma cells via both SMAD4-dependent and -independent pathways in head and neck cancer.

In the present study, we found a reduced expression of *TGFBR3* in oral cancer patients in both the TCGA-HNC dataset and clinical specimens in NCKU hospital. Furthermore, the low expression in *TGFBR3* mRNA was associated with a poor prognosis of these patients. Ectopic overexpression of *TGFBR3* decreased migration and invasion of oral cancer cells, while the silencing of *TGFBR3* had the opposite effects. Furthermore, *TGFBR3* overexpression reduced buccal lymph node metastasis without affecting primary tumor growth in an animal model. These results indicated that TGFBR3 is a tumor suppressor in oral cancer and that GIPC1 and ARRB2, both of which interact with the cytoplasmic domain of TGFBR3, differentially mediate TGFBR3 anti-cancer activity (Figure 8). We further identified ANG, a previously known tumor promotor that mediates TGFBR3-dependent suppression of migration and invasion of oral cancer cells.

In an effort to elucidate the mechanism underlying the down-regulation of *TGFBR3* in oral cancer cells, we identified an inverse relationship between the expression of *TGFB1* and *TGFBR3* using the TCGA-HNC database. Indeed, TGF-β1 treatments of OC-2 oral cancer cells significantly decreased *TGFBR3* mRNA and protein abundance and promoted cell migration and invasion. These observations are significant because an increase of TGF-β1 expression was frequently detected in many human cancer types, including oral cancer, and its increase correlated with cancer invasiveness, metastasis, and a poor prognosis [35]. Furthermore, overexpression of *TGFBR3* reduced the activation of TGF-β1-mediated SMAD signaling and aggressive cell behaviors in OC-2 oral cancer cells (Figure 4c,d). These results support the notion that while TGFBR3 is essential for TGF-β signaling, it is also a critical component of a negative-feedback loop for canonical TGF-β signaling to ensure a proper amplitude of the signaling.

We have previously shown that the addition of ANG results in an inhibition of scar formation and a significant reduction of secreted TGF-β1 of fibroblasts [36]. The current study also showed that TGFBR3 elevates secreted ANG (Figure 7a,b). Thus, TGFBR3-mediated increases in ANG partly account for TGFBR3-dependent inhibition of TGF-β signaling.

The TGFBR3-mediated inhibition of TGF- β signaling can act through ectodomain shedding-dependent and -independent pathways [32]. In the shedding-independent pathway, TGFBR3 interacts with the scaffolding proteins, GIPC1 and ARRB2, through its highly conserved, short cytoplasmic domain [9,32]. The binding of TGFBR3 to ARRB2 mediates its internalization [10,11], whereas the association of TGFBR3 with GIPC1 stabilizes TGFBR3 at the cell surface [12]. Consistent with these findings, we found an increase in TGFBR3 protein levels upon *ARRB2* silencing (Figure 5a,d). In SMAD4-positive OC-2 oral cancer cells, both ARRB2 and GIPC1 participate in TGFBR3-mediated inhibition of migration and invasion. Notably, only ARRB2 mediates TGFBR3-dependent induction of secreted ANG, although the underlying mechanism remains unknown.

In contrast, only the loss of *GIPC1* blocked TGFBR3-mediated inhibition of migration and invasion in SMAD4-deficient CAL-27 cells. These results highlighted a pivotal role of GIPC1 in TGFBR3-mediated suppression of oral cancer cells, regardless of the status of SMAD4. Accordingly, HNC patients with a concordant reduction of *GIPC1* and *TGFBR3* expression had significantly poor prognosis compared to those with high expression of both genes (Figure 5e). Thus, the detailed mechanism by which GIPC1 mediates TGFBR3-associated inhibition of oral cancer cell migration and invasion warrants further investigation (Figure 8).

The tumor microenvironment plays an important role in tumor progression. Consistent with a role in fine-tuning TGF-β1 in CAFs and endothelial cells, CM obtained from TGFBR3-overexpressing cancer cell culture exerted an inhibitory paracrine effect on the migration and invasion of CAFs, and on endothelial tube formation. Conversely, CM derived from TGFBR3-depleted cancer cells had cancer-promoting effects on these stromal cells. These data indicated that TGFBR3 exerts a suppressive effect on both tumor cells and their stromal cells.

Conflicting data have indicated an inconclusive role of ANG in the pathogenesis of oral cancer. Some studies showed that ANG is pro-tumorigenic, and their expression positively correlates with disease stages [37,38], while others found no correlation between ANG and oral cancer [39]. In our analyses of the HNC database, we found that *ANG* mRNA is significantly lower in HNC patients compared to the healthy individuals (Figure S8a). However, *ANG* mRNA expression does not correlate with the overall survival rates (Figure S8b). Moreover, a recent report indicated that the serum levels of ANG in healthy individuals vary, depending on gender, age, and genetic factors [40]. Thus, we propose that one needs to be cautious when using serum ANG levels as a potential marker for oral cancer.

ANG is a small ribonuclease. The cellular function of ANG is intriguing and largely attributes to its ability to transactivate ribosomal RNA in the nucleoli and tRNA cleavage activity [41]. Notably, it was recently demonstrated that ANG binds to its receptor Plexin-B2 and exerts mitogenic activity through multiple kinase pathways that include ERK, AKT, and RAC [42]. Thus, ANG could function in cell membrane, cytosol, and nucleoli. Moreover, modification of ANG affects its cellular activity [41]. These properties may partly explain why ANG has cytotoxic or proliferative activities, depending on the contexts and cell types [43,44]. By using anti-ANG antibodies and knockdown approaches, we demonstrated that blocking or loss of ANG abrogates TGFBR3-dependent anti-migration and anti-invasion activities. Furthermore, in *TGFBR3*-knockdown cells, ANG is sufficient to inhibit migration and invasion. Our data clearly demonstrated that ANG, induced by TGFBR3 and a well-established pro-angiogenic factor [45], is essential for TGFBR3-mediated suppression of migration and invasion in OC-2 cells. It will be interesting to dissect whether TGFBR3-induced ANG has effects on known ANG pathways.

We noted that CM derived from TGFBR3-overexpressed OC-2 cells inhibits tube formation of HUVECs. This result is in contrast to the observation that ANG, induced by TGFBR3, potently promotes angiogenesis. We hypothesize that other cytokines, whose expression is regulated by TGFBR3 manipulation, override ANG’s angiogenic activity. For example, both MCP-1 and CCL20 are powerful angiogenic factors [46,47] and are significantly down-regulated by TGFBR3 overexpression. Furthermore, other secreted TGFBR3-regulated molecules not included in the array may promote CM’s anti-cancer activity.

## 4. Conclusions

The current findings define a role for TGFBR3 in HNC as a suppressor of cell migration, invasion, and metastasis through both SMAD4-dependent and -independent pathways (Figure 8). Among several types of TGFB signaling inhibitors, ligand traps that sequester ligands from receptor binding has shown promise with less side effects in early clinical trials for prostate and gynecological cancers [48,49,50]. This study should facilitate the possibility of using TGFBR3-mediated tumor suppression for oral cancer treatment.

## 5. Materials and Methods

### 5.1. Reagents

All culture medium power, Trizol reagent, Lipofectamine 2000, OPTI-MEM, zeocin, and RT-qPCR reagents were from ThermoFisher Scientific (Grand Island, NY., USA). Endothelial growth medium 2 (EGM2) for growing endothelial cells was from Lonza Inc. (Allendale, NJ, USA). Oligonucleotide primers for DNA sequencing and RT-qPCR (Table S1) were from MDbio (Taipei, Taiwan). All the chemicals, anti-TGFBR3 antibodies, and SB431542 were from Sigma-Aldrich Co (St. Louis, MO, USA). CellTiter 96^®^ AQueous One Solution (MTS kit) was from Promega Corp (Madison, WI, USA). pLKO_AS2.zeo was from the National RNAi Core facility in Academia Sinica, Taiwan. The antibodies for GIPC1, ARRB2, and pan-Cytokeratin (pan-CK) were from Santa Cruz Biotechnology (Dallas, TX, USA). Phospho-SMAD Antibody Sampler Kit was from Cell Signaling Technology (Danvers, MA, USA). Matrigel was from BD Biosciences (San Jose, CA, USA). Millicell culture inserts were from Millipore EMD (Darmstadt, Germany).

### 5.2. Patient Specimens

Oral cancer specimens with clinicopathologic characteristics (Table S2) were from 86 treatment-naive patients (median age = 52) undergoing surgery at National Cheng Kung University (NCKU) hospital. With the patients’ informed consent, the biopsies of histologically proven healthy tissue in oral cavities other than tumor sites were taken as pair-wise normal controls. All the fresh samples were handled anonymously for fibroblast isolation or snap-frozen and stored in liquid nitrogen until use. This study was conducted after the approval of the Institutional Review Board at National Cheng Kung University (A-BR-101-038 and A-ER-104-389). All participants gave consent to participate in the study and for publication.

### 5.3. Cell Culture

Human oral cancer lines, OEC-M1 and OC-2, were propagated in RPMI-1640 medium containing 10% fetal bovine serum (FBS) supplemented with 100 units/mL penicillin and 100 μg/mL streptomycin. Human oral cancer line CAL-27 and HEK293T cells were maintained in DMEM medium containing 10% FBS supplemented with 100 units/mL penicillin and 100 μg/mL streptomycin. Two additional oral cancer lines, SAS and SCC-9, were propagated in DMED/F12 with 10% FBS, 1% penicillin/streptomycin solution and 0.4 μg/mL hydrocortisone. Human umbilical vein endothelial cells (HUVECs) were isolated with informed consent and maintained as previously described [51]. Before the use, all the oral cancer lines were validated for their cell identity by short tandem repeat profiling analysis.

### 5.4. Human TGFBR3 Expression Construct

The Myc/FLAG-tagged human TGFBR3 cDNA was cloned by PCR and subcloned into the pLKO-AS2.zeo lentiviral vector for stable expression in the cells of interest.

### 5.5. Gene Silencing

All shRNA clones or shLuc were expressed in the lentiviral vector. The control Luc shRNA were purchased from the National RNAi Core Facility (Academia Sinica, Taipei, Taiwan). The shRNA sequence and clone numbers were listed in Table S3. The lentivirus packaging, infection, and selection were performed as described by the core facility. The indicated plasmids were introduced into the cells by using Lipofectamine 2000 following the manufacture’s protocol.

### 5.6. Xenograft Transplantation and Buccal Metastasis

Male BALB/cAnN.Cg-*Foxn1^nu^*/CrlNarl mice (6–8 weeks old) were purchased from the National Laboratory Animal Center in Taiwan. The use of these animals and experimental protocols were reviewed and approved by the Institutional Animal Care and Use Committee (IACUC) at NCKU. All animal experiments complied with the ARRIVE guides and were performed in accordance with the National Institutes of Health Guide for care and use of laboratory animals (NIH publications No.8023, revised 1978). For xenograft transplantation, vector control or TGFBR3-overexpressing CAL-27 or OC-2 cells (2 × 10^6^ cells) together with 50 μg Matrigel were subcutaneously injected into mouse flanks (5 mice in each group). One week after injection, tumor size was measured every 2 days for the indicated time until ending points. For buccal metastasis, 7~8 mice per group were orthotopically injected with TGFBR3-overexpressing OC-2 cells into buccal mucosa. At ~7 weeks post-implantation, primary tumors and cervical lymph nodes were collected from the mice and embedded with paraffin after euthanization. We subjected these tissue sections to H&E staining for histological examination. Metastatic tumor cells in the lymph nodes were stained with an anti-pan-CK antibody, a marker for epithelial cells.

### 5.7. Preparation of Conditioned Medium (CM)

Subconfluent cells were refed with serum-free culture medium 1 day after seeding. CM was harvested after 24-h incubation and centrifuged at 3000 rpm to remove cell debris. We used Vivaspin 6 columns (5 kDa MWCO, GE Healthcare, Piscataway, NJ, USA) to concentrate the CM.

### 5.8. Endothelial Tube Formation Assay

Human umbilical vein endothelial cells (HUVECs) were seeded at 2 × 10^4^ per well in duplicate onto 48-well culture dishes coated with 100 μL of Matrigel (13.4 mg/mL) and subjected to CM treatment. Tube formation was photographed at 6 hours post-seeding with an inverted Olympus CKX31 phase-contrast microscope (Tokyo, Japan). The branch point number in four random high-power fields (40× magnification) was quantified by the imaging software developed by Dr. YN Sun at National Cheng Kung University.

### 5.9. TGF-β1 and Drug Treatment

The indicated cells were serum-deprived for 24 h and then treated for recombinant human TGF-β1 (R&D Systems, Minneapolis, MN, USA) at the indicated doses before the isolation of total RNA or protein for subsequent analyses. For pharmacological inhibition, serum-derived cells were pre-treated for 1 hour with DMSO (vehicle control) or 10 μM SB431542 (TGFBR1 inhibitor) prior to the stimulation of TGF-β1 (10 ng/mL) for 30 min.

### 5.10. Cytokine Array

We measured in the CM the level of 120 cytokines as described by C-Series Human Cytokine Antibody Array 1000 Kit, consisting of both Human Cytokine Antibody Array C6 and C7 (RayBiotech Inc. Norcross, GA, USA). Following array blockage with the blocking buffer for 30 min, we incubated the blocked array with 1 mL of CM overnight at 4 °C. The hybridized array was washed three times with Wash Buffer I, and two times with Wash Buffer II at room temperature. We then added biotin-conjugated C6 primary antibodies to the washed array. Following overnight incubation at 4 °C and washes, we added diluted HRP-conjugated streptavidin. After overnight incubation at 4 °C and washes, the array was developed by using ECL, and proteins were detected and quantitated by FluorChem HD2 systems (ProteinSimple, Santa Clara, CA, USA). Cytokines levels were quantified against internal controls in the array by using Image J software and expressed as fold changes following the comparison with control samples.

### 5.11. ANG ELISA Assay

The level of angiogenin protein in the CM was quantified in triplicate by using Human Angiogenin DuoSet ELISA assay (R&D Systems, Minneapolis, MN, USA).

### 5.12. Antibody Neutralization

For ANG neutralization studies, the anti-ANG antibody at the indicated concentration was added onto the treatment medium prior to its use for treating the indicated cells for cell migration and invasion assays.

### 5.13. Statistical Analysis

All analyses were performed by using the statistical software SPSS for Windows Version 17 (SPSS Inc., Chicago, IL, USA). Data were represented as mean ± SD or SEM. Biological repeats for cell-based studies were statistically analyzed by the Two-tailed Student’s *t*-test. Linear regression and Pearson correlation were used to assess the relationship between gene expression. The Chi-square test was used to compare two sample rates. The Kaplan–Meier method and log-rank test were used to compare the survival among patient groups. Significance was set at *p* < 0.05.

## Supplementary Materials

The following are available online at https://www.mdpi.com/2072-6694/12/6/1375/s1, Table S1: List of primers for PCR and the mutation, Table S2. Clinicopathologic characteristics of 86 oral cancer patients in NCKU cohort, Table S3. shRNA clones used in the gene silencing experiments, Figure S1: The decrease of TGFBR3 protein expression in oral cancer relative to adjacent normal tissue, Figure S2: Differential expression of TGFBR3 and SMAD proteins in oral cancer cells, Figure S3: A summary of genetic alterations of TGFBR3 gene in HNC patients, Figure S4: The impact of ARRB2 mRNA expression or its relation with TGFRB3 on the clinical outcome of TCGA-HNC patients, Figure S5: Characterization of adjacent normal fibroblasts (NFs) and cancer-associated fibroblasts (CAFs) isolated from human oral cancer tissue samples, Figure S6: CM derived from vector or TGFRB3-expressing OC2 cells had no effect on the proliferation of ECs or CAFs, Figure S7: Ectopic expression of TGFBR3 increased secreted ANG protein levels in SMAD4-positive 293T cells, Figure S8: The deregulation of ANG mRNA has no impact on TCGA-HNC patient clinical outcomes, Figures S9–S21: uncropped blots.
