# Supplementary material for: The Tumor Suppressor TGFBR3 Blocks Lymph Node Metastasis in Head and Neck Cancer
Source: Cancers (Basel). 2020 May 27;12(6):1375. doi: 10.3390/cancers12061375 (PMC7352722; doi:10.3390/cancers12061375)

The tumor suppressor TGFBR3 blocks lymph node metastasis in head and neck cancer

Wei-Yu Fang[1], Yi-Zih Kuo[2], Jang-Yang Chang[3,4], Jenn-Ren Hsiao[2], Hung-Ying Kao[5], Sen-Tien Tsai[2], and Li-Wha Wu[6,7]

[1]Institutes of Basic Medical Sciences, College of Medicine, National Cheng Kung University, Tainan 70101, Taiwan, R.O.C.
[2]Department of Otolaryngology, National Cheng Kung University Hospital, College of Medicine, National Cheng Kung University, Tainan 70101, Taiwan, R.O.C.
[3]National Institute of Cancer Research, National Health Research Institutes, Tainan 70456, Taiwan, R.O.C.
[4]Division of Hematology/Oncology, Department of Internal Medicine, National Cheng Kung University Hospital, College of Medicine, National Cheng Kung University, Tainan 70101, Taiwan, R.O.C.
[5]Department of Biochemistry, School of Medicine, Case Western Reserve University, Cleveland, OH 43210, U.S.A
[6]Institute of Molecular Medicine, College of Medicine, National Cheng Kung University, Tainan 70101, Taiwan, R.O.C.
[7]Department of Laboratory Science and Technology, College of Health Sciences, Kaohsiung Medical University, Kaohsiung 80708, Taiwan, R.O.C.

Please address correspondence to:
Li-Wha Wu, Institute of Molecular Medicine, College of Medicine, National Cheng Kung University, Tainan 70101, Taiwan, R.O.C. Tel:+886-6-2353535 ext. 3618; Fax:+886-6-2095845; E-mail: liwhawu@mail.ncku.edu.tw; Sen-Tien Tsai, Department of Otolaryngology, National Cheng Kung University Hospital, College of Medicine, National Cheng Kung University, Tainan 70101, Taiwan, R.O.C. Tel:+886-6-2353535 ext.5311; E-mail: T602511@mail.ncku.edu.tw.

Supplementary information

Methods

IHC staining

Patient tissue sections (5-μm thickness) were deparaffinized through gradient alcohol and xylene. Hematoxylin and eosin staining were used to confirm the original histopathological diagnosis. Following quenching peroxidase by hydrogen peroxide,

we employed an antigen retrieval method to enhance the immunodetection before antibody incubation. Consecutive tissue sections from the same patient were individually incubated overnight with the indicated antibodies at 4°C followed by incubation with the secondary antibody. The immunocomplexes were detected by the Dako REAL™ EnVision™ Detection System, Peroxidase/DAB+ (Hamburg, Germany).

Isolation and cultivation of oral CAFs and NFs

Briefly, fresh tissues were washed several times with phosphate-buffered saline (PBS) and antibiotics. The tissues were sliced into small pieces (1 × 1 × 1 mm) and maintained in DMEM containing 20% FBS, glutamine (20 μg/ml), penicillin (100 U/ml), and streptomycin (100 μg/ml) at 37°C with 5% CO_2. Following the cellular characterization of the identity of these isolated cells at passage 3, isolated NFs and CAFs at 5 to 7 passages were used for the subsequent studies.

RNA isolation and RT-qPCR

Total RNA was isolated by using TRIzol reagents from the indicated cells or snap-frozen tissues. One μg RNA was reverse-transcribed into cDNA using the High Capacity cDNA Reverse Transcription Kit. We amplified cDNA samples by using the Fast SYBR Green Master Mix and determined the cycle threshold (Ct), the fractional cycle number at which the amount of an amplified target reaching a fixed threshold. The mRNA expression of the indicated genes in triplicates was calculated by using $2^{-\Delta Ct}$ ($\Delta Ct = Ct^{target\ gene} - Ct^{28S\ rRNA}$). The primers were listed in Table S1.

Nuclear and cytosol fractionation

Following the TGF-β stimulation (10 ng/mL) for one hour, the indicated cells were washed with phosphate-buffered saline and harvested in lysis buffer (10 mM Tris-HCI, pH 6.8, 10 mM NaC1, 3 mM $MgC1_2$, 0.05% NP-40, 1 mM EGTA, 1 mM Na_3VO_4, 50 mM NaF, and 1 nM okadaic acid) containing protease inhibitors. Following lysate centrifugation at 20,800 g for 15 min at 4 °C, the supernatant was collected for the cytosolic fraction. The pellet was subsequently washed with a wash buffer (10 mM PIPES, pH 6.8, 25 mM NaC1, 3 mM $MgC1_2$, 300 mM sucrose, 1 mM EGTA, 1 mM Na_3VO_4, and 50 mM NaF) followed by centrifugation at 2,700 g for 5 min at 4 °C. The pellet was resuspended in 100 μL of wash buffer and layered with a 1 ml sucrose buffer (1M Sucrose, 1 mM Na_3VO_4, and 50 mM NaF) followed by centrifugation at 2,700 g for 10 min at 4 °C. The resulting pellet was washed with the wash buffer and then extracted with an extraction buffer (20 mM HEPES, pH 7.9, 300 mM NaC1, 1.5 mM $MgC1_2$, 0.2 mM EDTA, 1 mM Na_3VO_4, 0.1 mM β-glycerophosphate, 50 mM NaF, and 1 nM okadaic acid) on ice for 30 min. After centrifugation at 20,800 g for 15 min at 4

°C, the supernatant was stored as a nuclear extract for subsequent studies.

Western Blot analysis

Cells were lysed in the lysis buffer (50 mM HEPES pH 7.4, 150 mM NaCl, 1% Triton X-100, 10% glycerol, 1 mM EGTA, 1 mM EDTA, 10 mM sodium pyrophosphate, 100 mM sodium fluoride, 0.2 mM sodium orthovanadate) with a protease inhibitor cocktail (Biotool, Houston,TX, USA). Following centrifugation at 13,000 rpm for 15 minutes to remove cell debris, we measured the protein concentration by Bio-Rad Bradford Protein Assays (Hercules, CA, USA). Equal amounts of total protein were subjected to SDS-PAGE, followed by Western blots probed with the indicated antibodies, and detected by Chemiluminescence Reagent. Densitometry was used to quantify the expression of the indicated protein.

Cell proliferation assay

Two different assays, cell enumeration and OD_{492} measurement by MTS kits, were used for measuring cell proliferation. For cell enumeration, the indicated cells were seeded in triplicate at 10-20% confluence in 24-well plates. Cells were harvested for viable cell count by trypan blue exclusion on a daily basis for four days after seeding. For OD_{492} measurement, the indicated cells were seeded in quadruplicate in 96-well plates and subjected to growth in a CO_2 incubator for two days prior to the use of MTS kits. This experiment was independently repeated three times. Data are mean ± SD.

Wound repair assay

Culture dishes (35 mm), coated overnight with type I collagen (5 μg/mL) from rat tails, were seeded with 90% confluent cell density in the growth medium. After 16 hours, cells were treated with mitomycin C for 24 hours. Cell monolayers were wounded by scraping with a pipette tip and incubated at 37°C with the CM if needed. Cell migration was monitored and photographed at the indicated time post-wounding. The mean distance of ten wound widths along the wound before and after the migration was calculated. The migration rate was the cell migration distance per hour and expressed as Mean ± SD. This experiment was independently repeated three times.

Invasion assay

Invasion assays were performed in 24-well Transwell units with 8-μm-pore polycarbonate membranes. The indicated cells ($3x10^5$ cells per well for cancer cells or $3x10^4$ cells per well for CAFs) in 250 μL of the starvation medium were added in duplicate onto upper chambers, precoated with 1 mg/mL Matrigel for cancer cells or 2 mg/mL collagen for CAFs. Lower chambers were filled with 500 μL growth medium

or CM. After 24-h (for cancer cell) or 48-h (for CAFs) incubation, cells that remained attached to the upper side of the filter were removed with cotton swabs. Cells that had migrated through the membrane to the lower surface were stained with Giemsa solution and counted in five random fields under a light microscope at 100X or 200X magnification. Each experiment was repeated three times, and results were expressed as mean ± SD.

Table S1. Clinicopathologic characteristics of 86 oral cancer patients in NCKU cohort

Table S2. List of primers for PCR and the mutation

Table S3. shRNA clones for gene silencing

Supplementary figure legends

Figure S1. The decrease of TGFBR3 protein expression in oral cancer relative to adjacent normal tissue. Following IHC staining, the stained tissue image of another representative oral cancer was taken under 400 x magnification. Scale bar, 100 μm.

Figure S2. Differential expression of TGFBR3 and SMAD proteins in oral cancer cells. a TGFBR3 protein expression in five oral cancer cell lines by Western Blot analysis. Actin was a loading control. **b** Western blot analysis of SMAD4 protein expression in the indicated oral cancer cell lines. Little or no SMAD4 was detected in the SMAD4-null CAL-27 cells. Actin was a loading control. All the uncropped blots with molecular weight markers for Figure S2a and S2b are shown in Figure S19.

Figure S3. A summary of genetic alterations of *TGFBR3* gene in HNC patients. Shown are OncoPrint outputs (cBioPortal for Cancer Genomics; www.cbioportal.org), where each bar represents a tumor found to contain a DNA alteration (amplification or mutation, as indicated).

Figure S4. The impact of ARRB2 mRNA expression or its relation with TGFRB3 on the clinical outcome of TCGA-HNC patients. Kaplan-Meier analysis showing the relation of overall survival with the expression of ARRB2 (Left) and ARRB2/TGFBR3 (Right) in the TCGA-HNC dataset.

Figure S5. Characterization of adjacent normal fibroblasts (NFs) and cancer-associated fibroblasts (CAFs) isolated from human oral cancer tissue samples. a A representative image of cell morphology of NFs and CAFs. **b** Western blot analysis was used to analyze the expression of Pan-CK (an epithelial cell marker), vimentin (a fibroblast marker), FSP-1 (a fibroblast marker), and α-SMA (a CAF marker). All the uncropped blots with molecular weight markers for Figure S5b are shown in Figure S20.

Figure S6. CM derived from vector or TGFRB3-expressing OC2 cells had no effect on the proliferation of ECs or CAFs. Following 48-hour treatment of TGFBR3-vector or overexpressing OC-2 cells with the indicated CM, the numbers of CAFs and ECs were measured by MTS kits and presented as mean ± SD. N.S., not significant versus vector-CM.

Figure S7. Ectopic expression of TGFBR3 increased secreted ANG protein levels in SMAD4-positive 293T cells. Left, Western blot analysis of 293T cells transfected with TGFBR3-bearing expression vector. Actin was a loading control. Right, we used ELISA to analyze the release of ANG in the CM from vector or TGFBR3-expressing 293T cells. Results are expressed as the mean pg/ml ± SD. The uncropped blots with molecular weight markers are shown in Figure S21.

Figure S8. The deregulation of *ANG* mRNA has no impact on TCGA-HNC patient clinical outcomes. a An Oncomine analysis of *ANG* mRNA expression in 3 HNC patient cohorts. We used box-plot diagrams to compare the mRNA levels of ANG in normal tissues with those in tumor tissues using Oncomine datasets. **b** The overall survival rates of HNC patients (N=497) were analyzed using the Kaplan-Meier curve and log-rank test based on high (> median) and low (< median) mRNA levels for *ANG* or *TGFBR3/ANG* from the TCGA cohort.

Table S1. List of primers for PCR and the mutation

Gene	Primer sequence
TGFB1 (qPCR)	F: 5’-TGGCGATACCTCAGCAACC-3’
	R: 5’-GACAGCTGCTCCACCTTGG-3’
TGFBR3 (qPCR)	F: 5’-GGAGGTGCATGTCC-TGAATC-3’
	R: 5’-CAGACTTGTGGTGGATGTGG-3’
ANG (qPCR)	F: 5’-ACTCCAGG-TACACACACTTCC-3’
	R: 5’-TGATGTCTTTGCAGGGTGAG-3’
SMAD4 binding site (ChIP-qPCR)	F: 5’-CTCCATTCCACACCCTCTCC-3’
	R: 5’-GCTCCCTGATGTCCTCACTT-3’
SMAD4 binding site (Mutation)	F: 5’-CCACACCCTCTCCCTCCCGT**AAAAA**TGGA-CGTGTAAGCGGAAGAG-3’
	R: 5’-CTCTTCCGCTTACACGTCCA**TTTTT**ACGG-GAGGGAGAGGGTGTGG -3’

Table S2. Clinicopathologic characteristics of 86 oral cancer patients in NCKU cohort

	Number of cases	% of total
**Median age (y)**		
＜52	42	48.8
≧52	44	51.2
**Tumor site**		
Buccal + Tongue	69	80.2
Others	17	19.8
**Stage**		
I + II	30	34.9
III + IV	56	65.1
**Tumor status (T)**		
T1 + T2	54	62.8
T3 + T4	32	37.2
**Lymph nodes (N)**		
No	42	48.8
Yes	44	51.2
**Distant metastasis (M)**		
No	85	98.8
Yes	1	1.2
**Differentiation**		
Well	44	51.2
Moderate + Poor	42	48.8
**Recurrence**		
No	63	73.3
Yes	23	26.7

Table S3. shRNA clones used in the gene silencing experiments

Target Gene	Clone number	Clone ID	Target Sequence
TGFBR3	#1	TRCN0000359081	GGAGTTGGTAAAGGGTTAATA
	#2	TRCN0000359000	TAATGGATTTCCGGGAGATAT
GIPC1	-	TRCN0000036769	GCAAATGCAATAATGCCCTCA
ARRB2	-	TRCN0000159332	GCTAAATCACTAGAAGAGAAA
ANG	#1	TRCN0000049663	TGCTGTCCTTGCCTTCCATTT
	#2	TRCN0000049666	ACGTTGTTGTTGCTTGTGAAA

Figure S1 by Fang WY et al

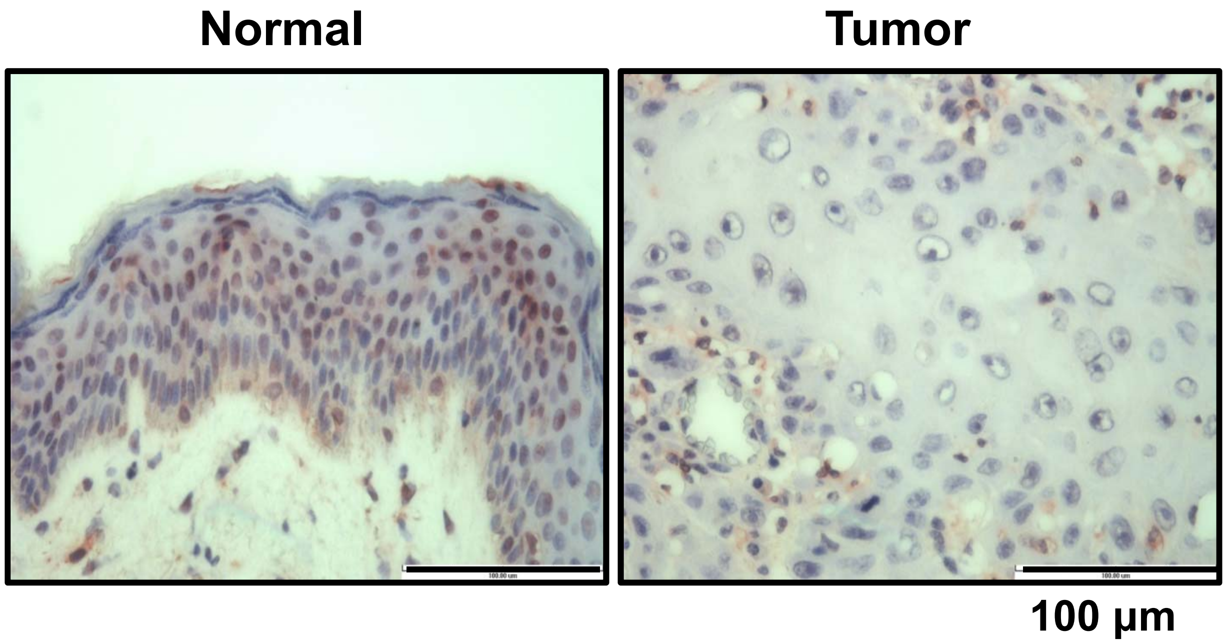

Figure S2 by Fang WY et al

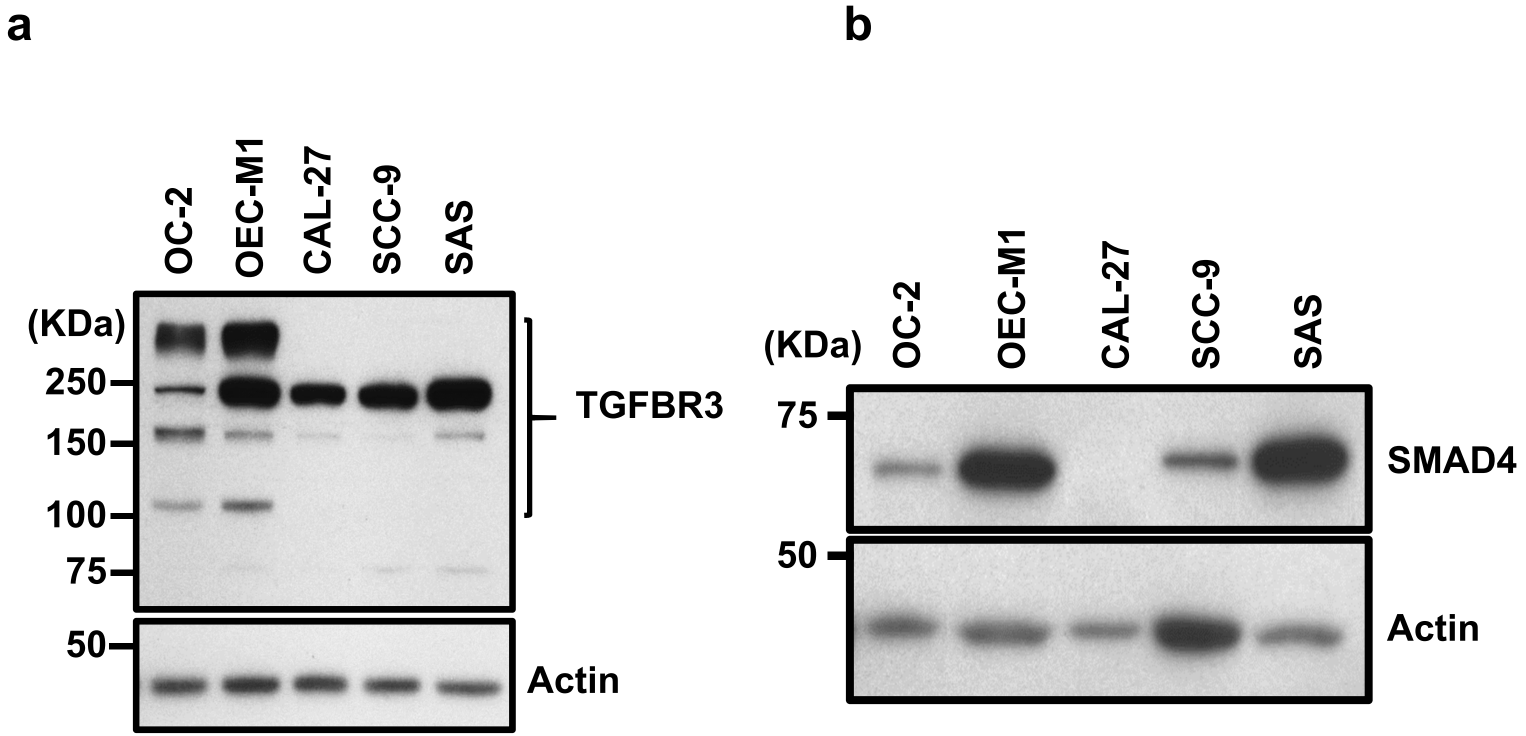

Figure S3 by Fang WY et al

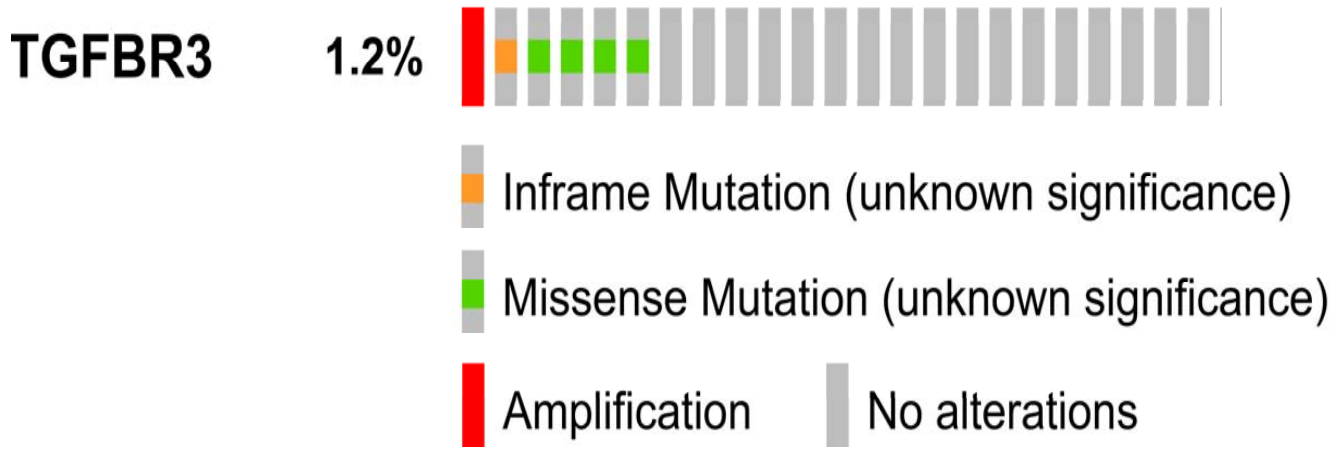

Figure S4 by Fang WY et al

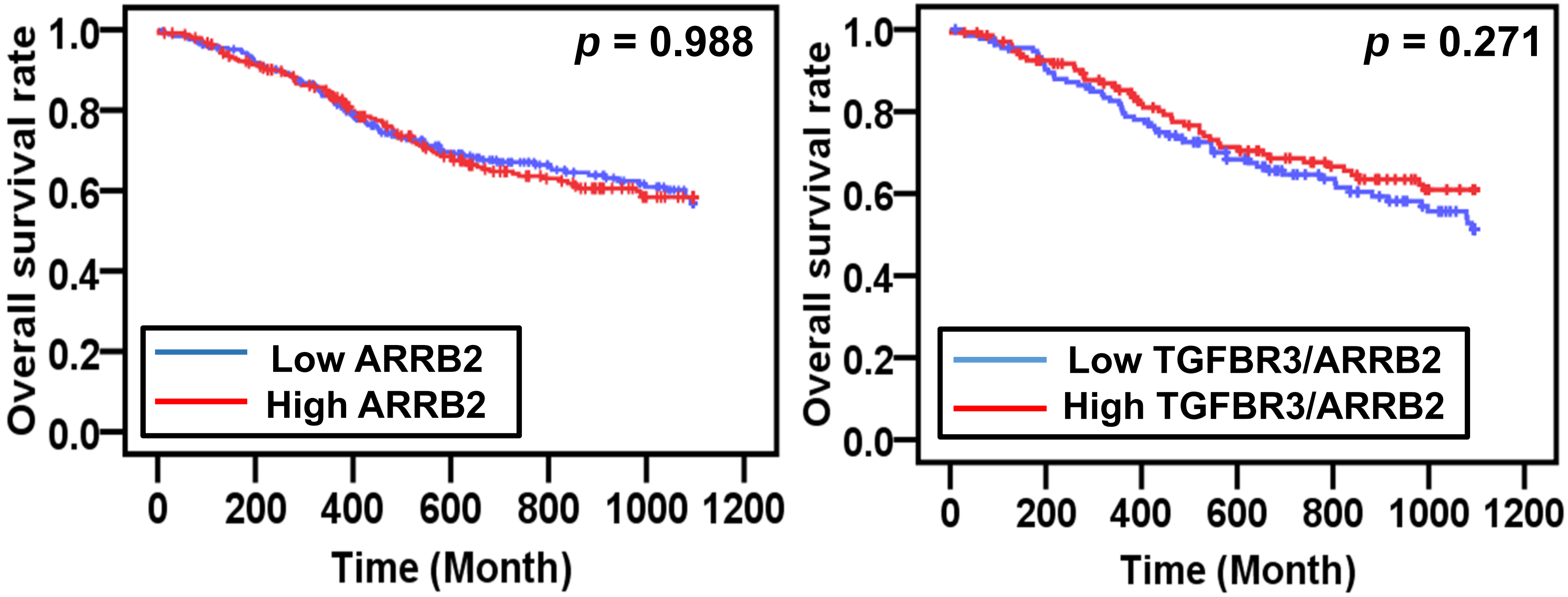

Figure S5 by Fang WY et al

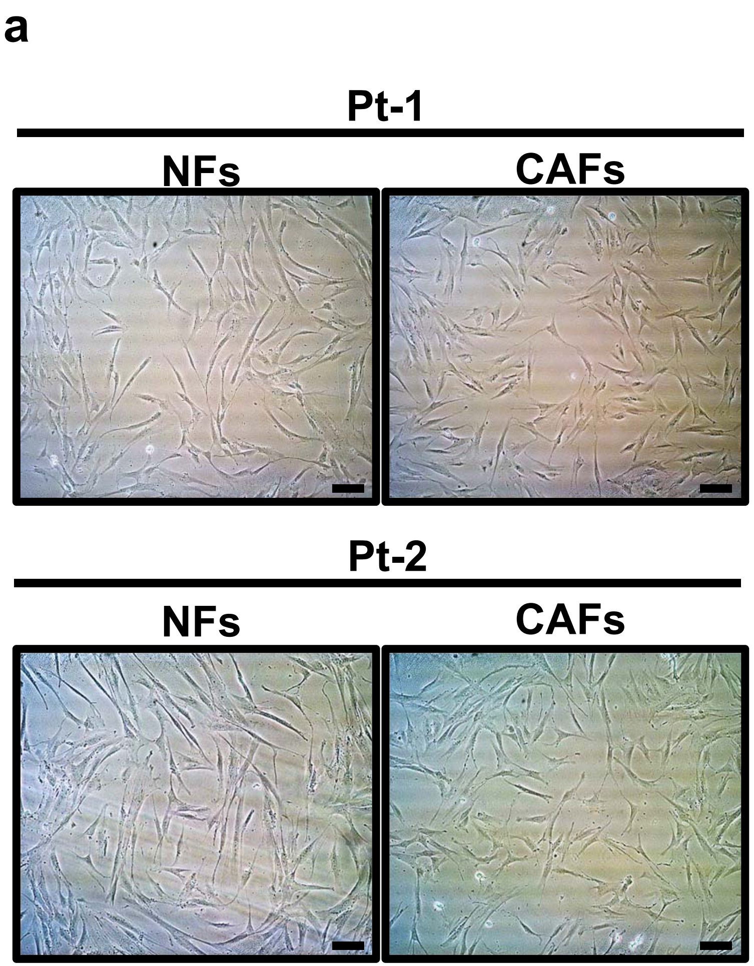

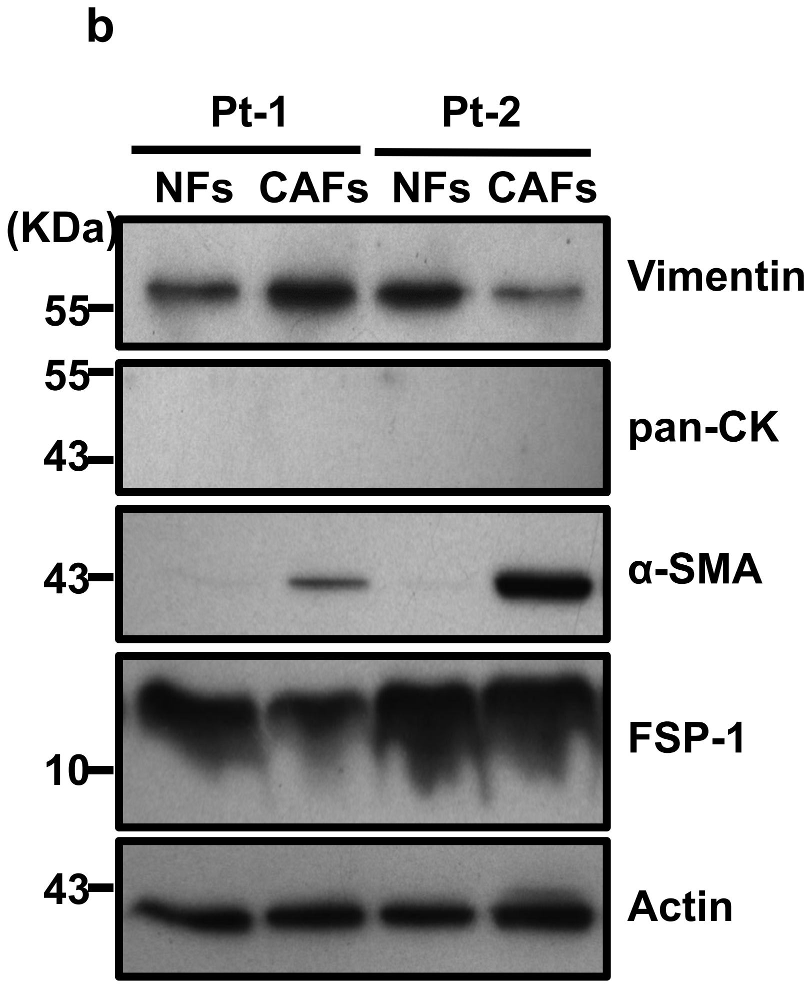

Figure S6 by Fang WY et al

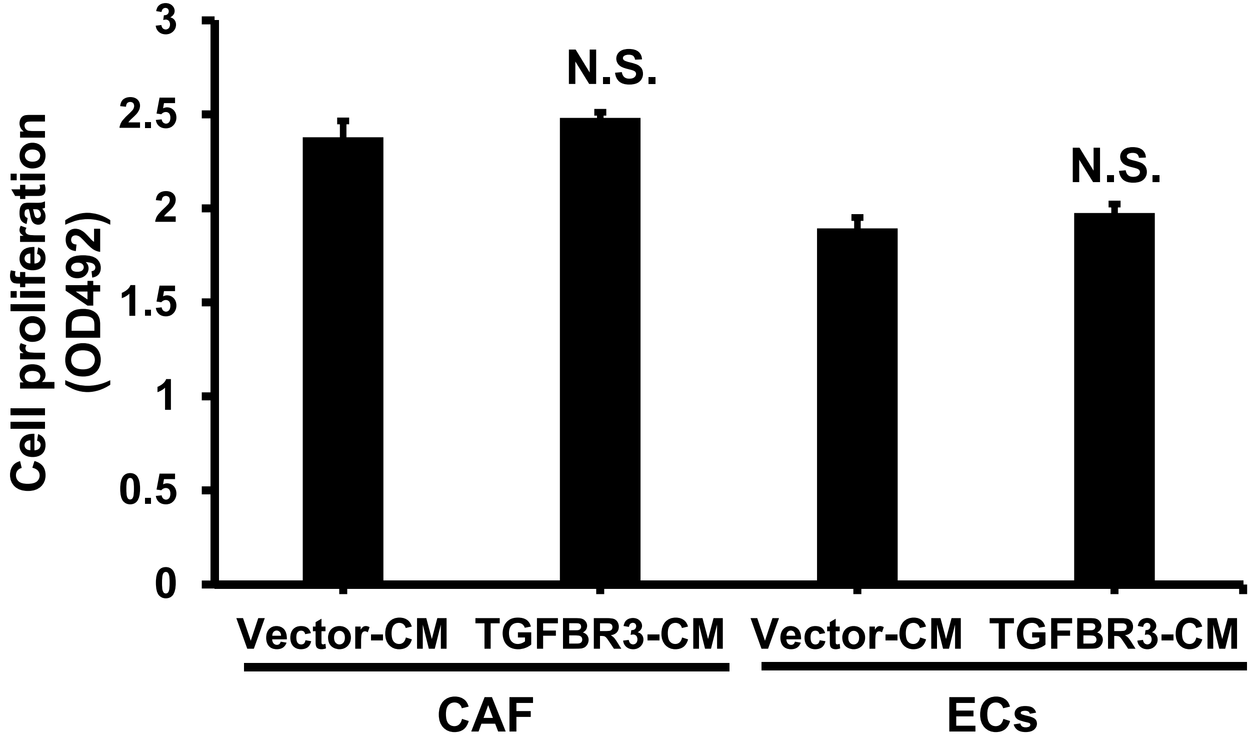

Figure S7 by Fang WY et al

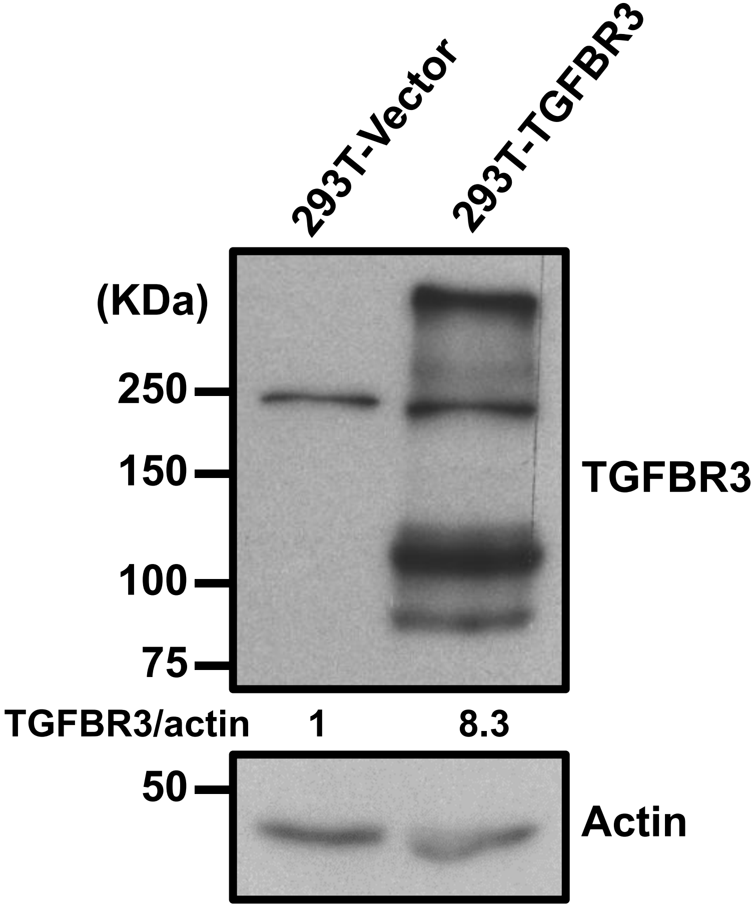

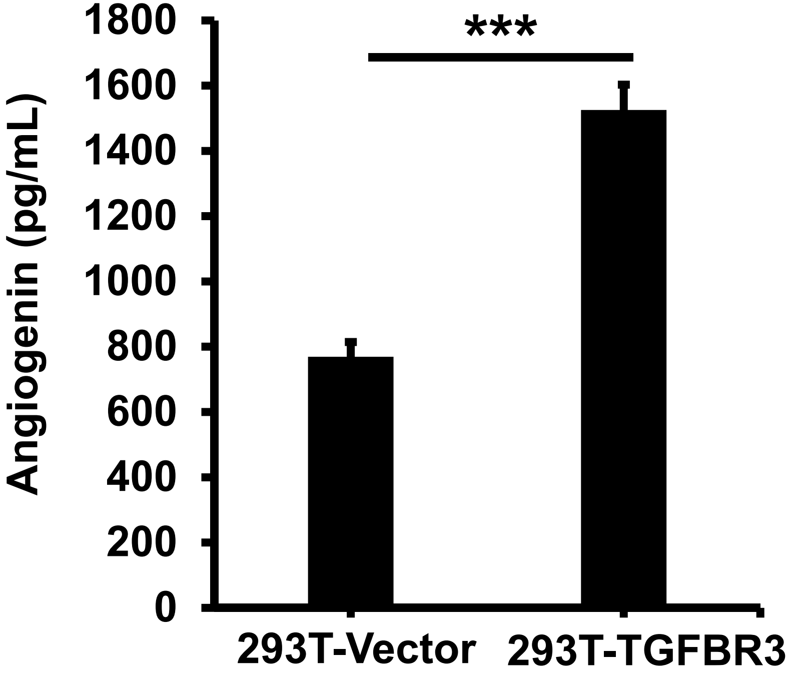

Figure S8 by Fang WY et al

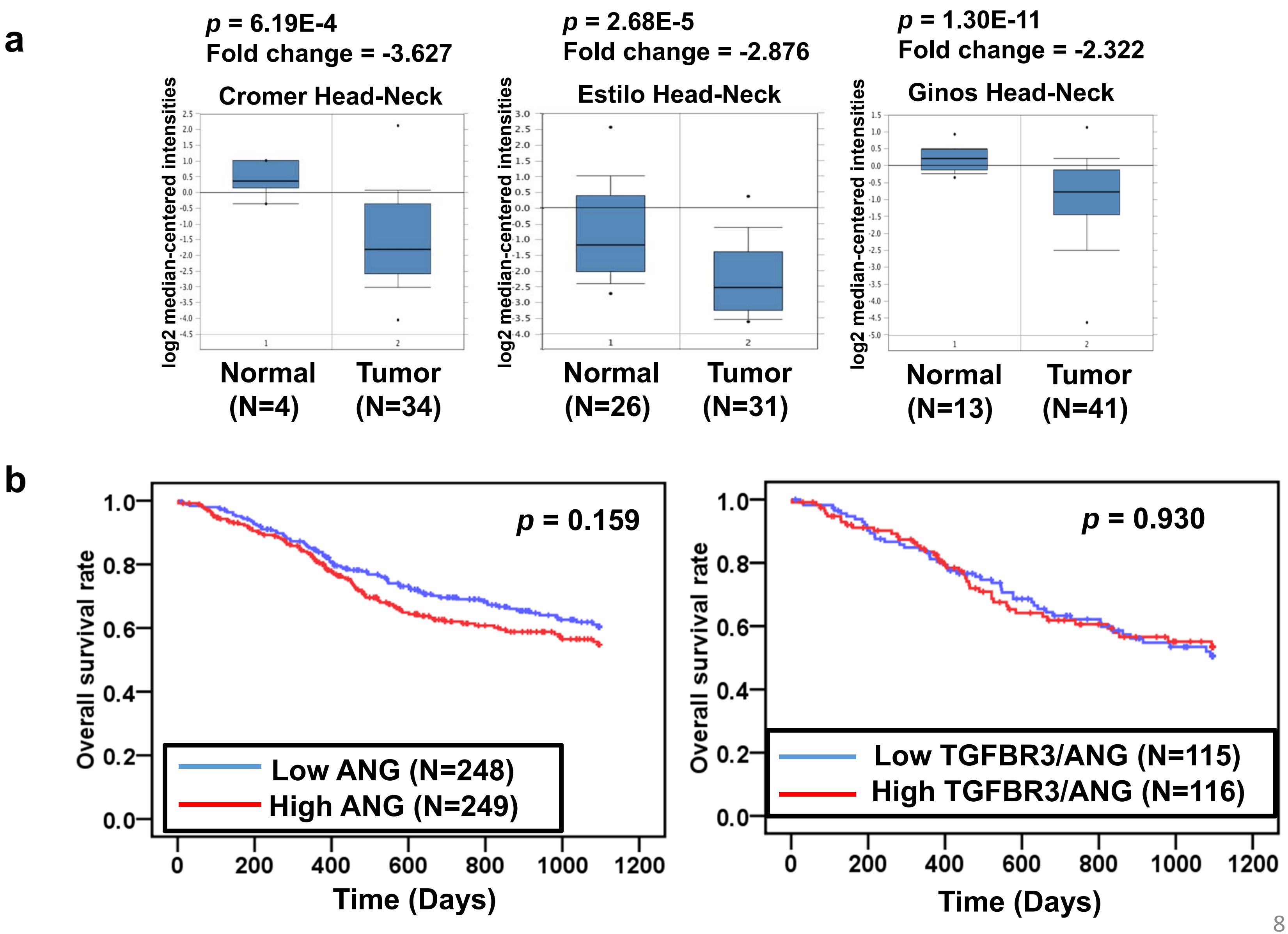

Figure S9 by Fang WY et al

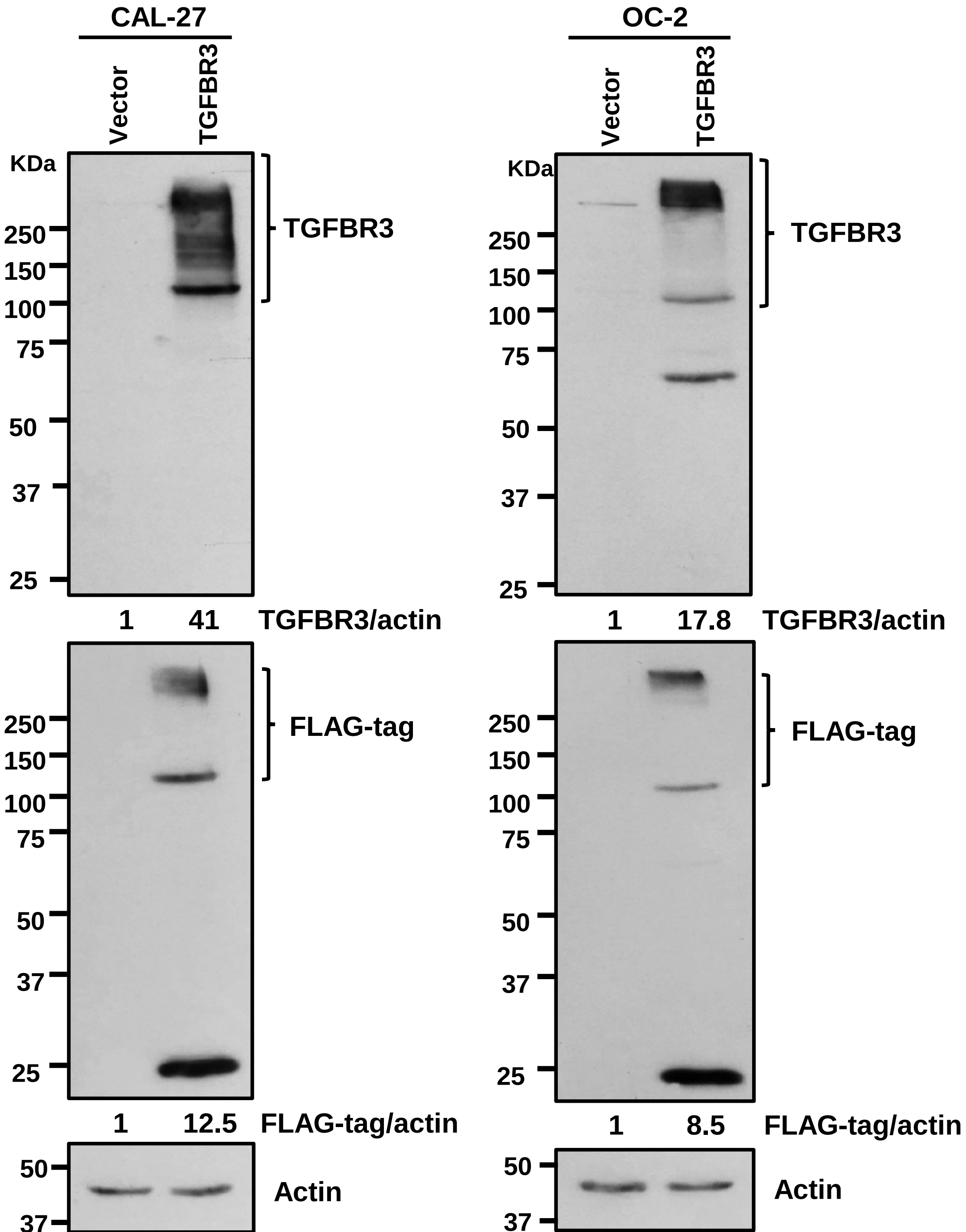

Figure S10 by Fang WY et al

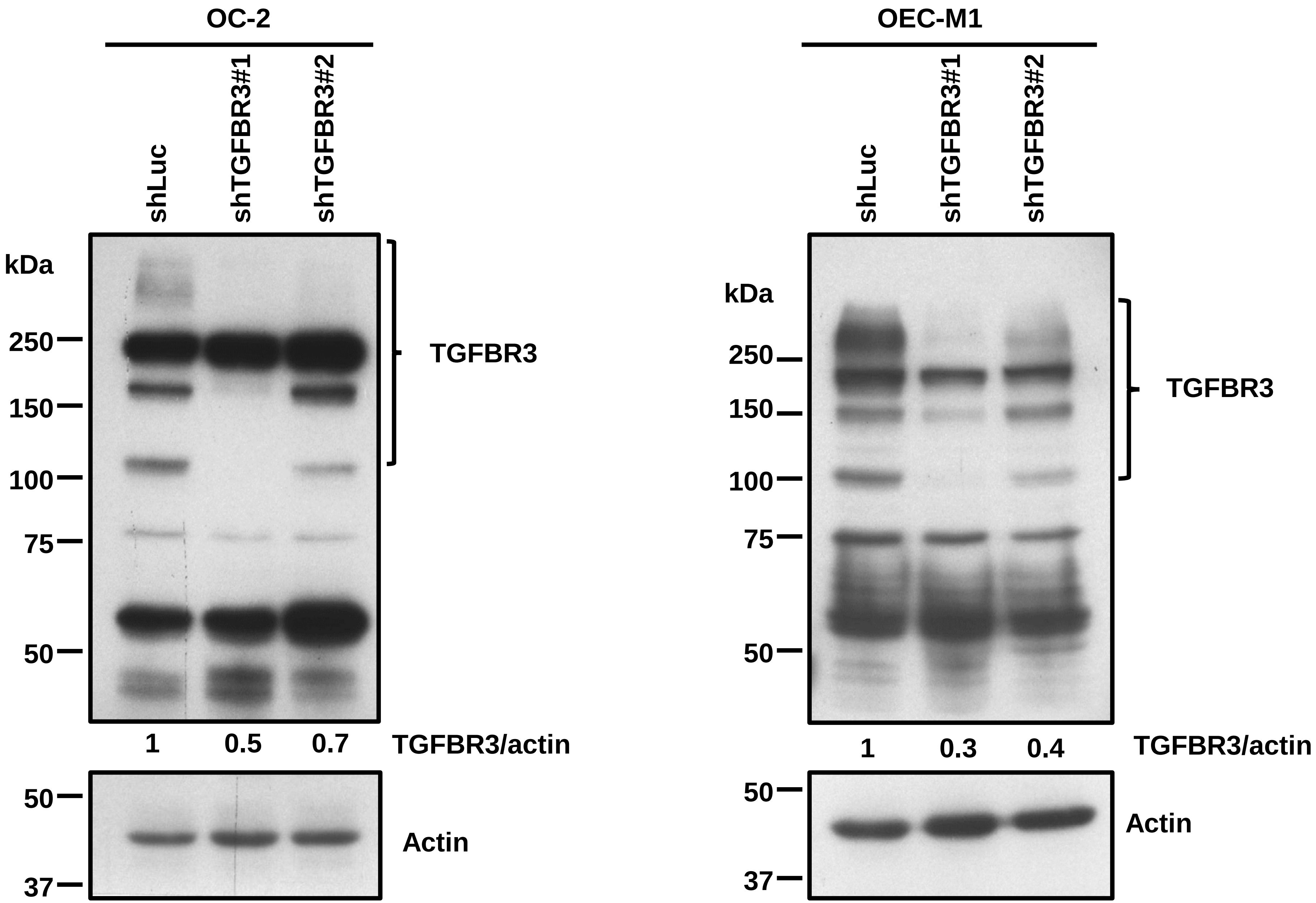

Figure S11 by Fang WY et al

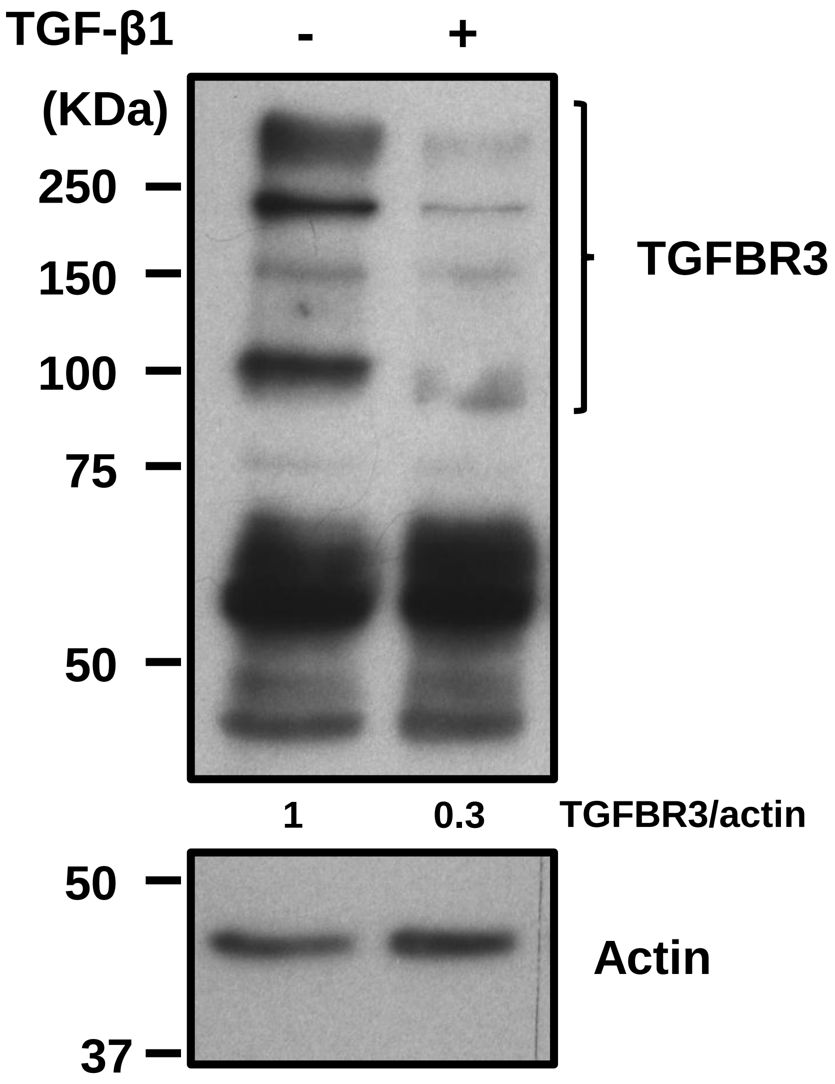

Figure S12 by Fang WY et al

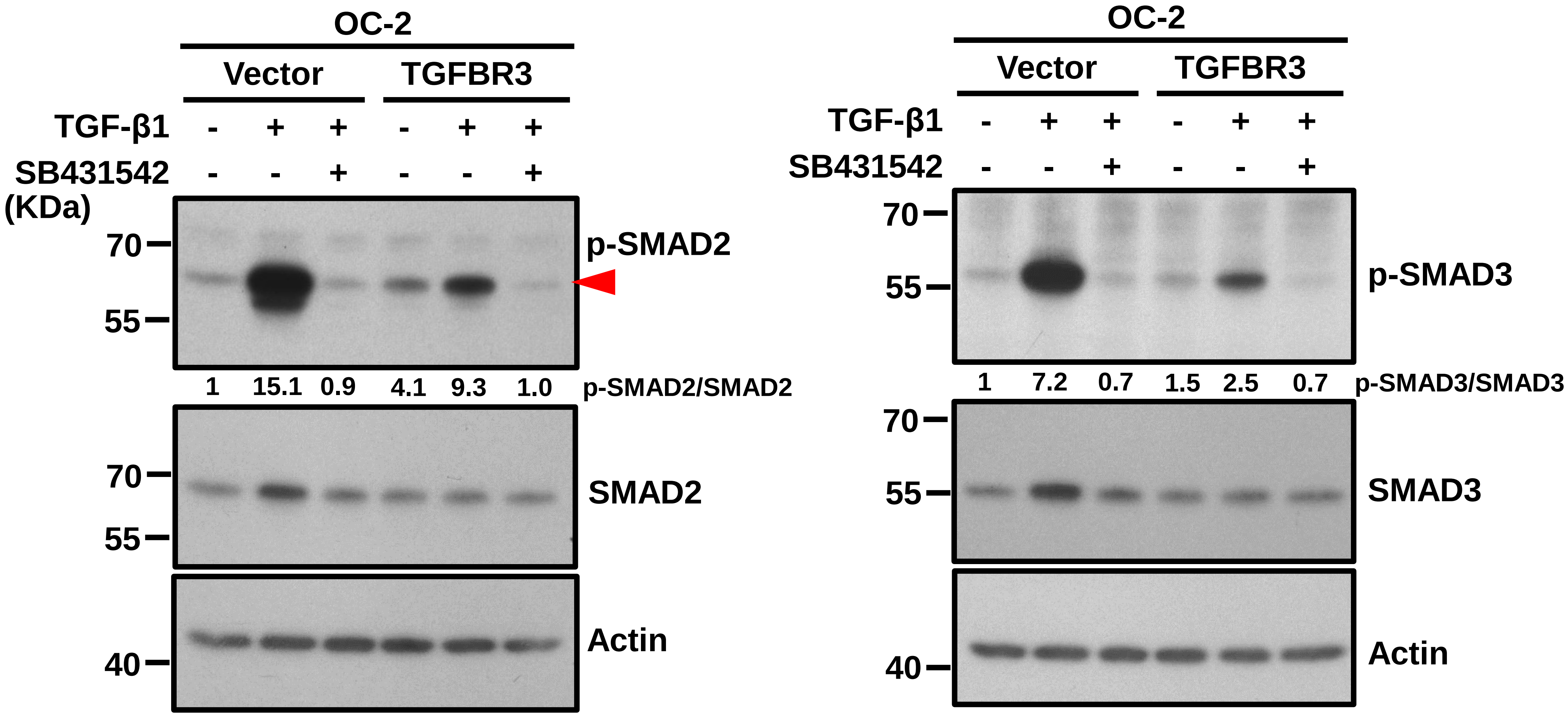

Figure S13 by Fang WY et al

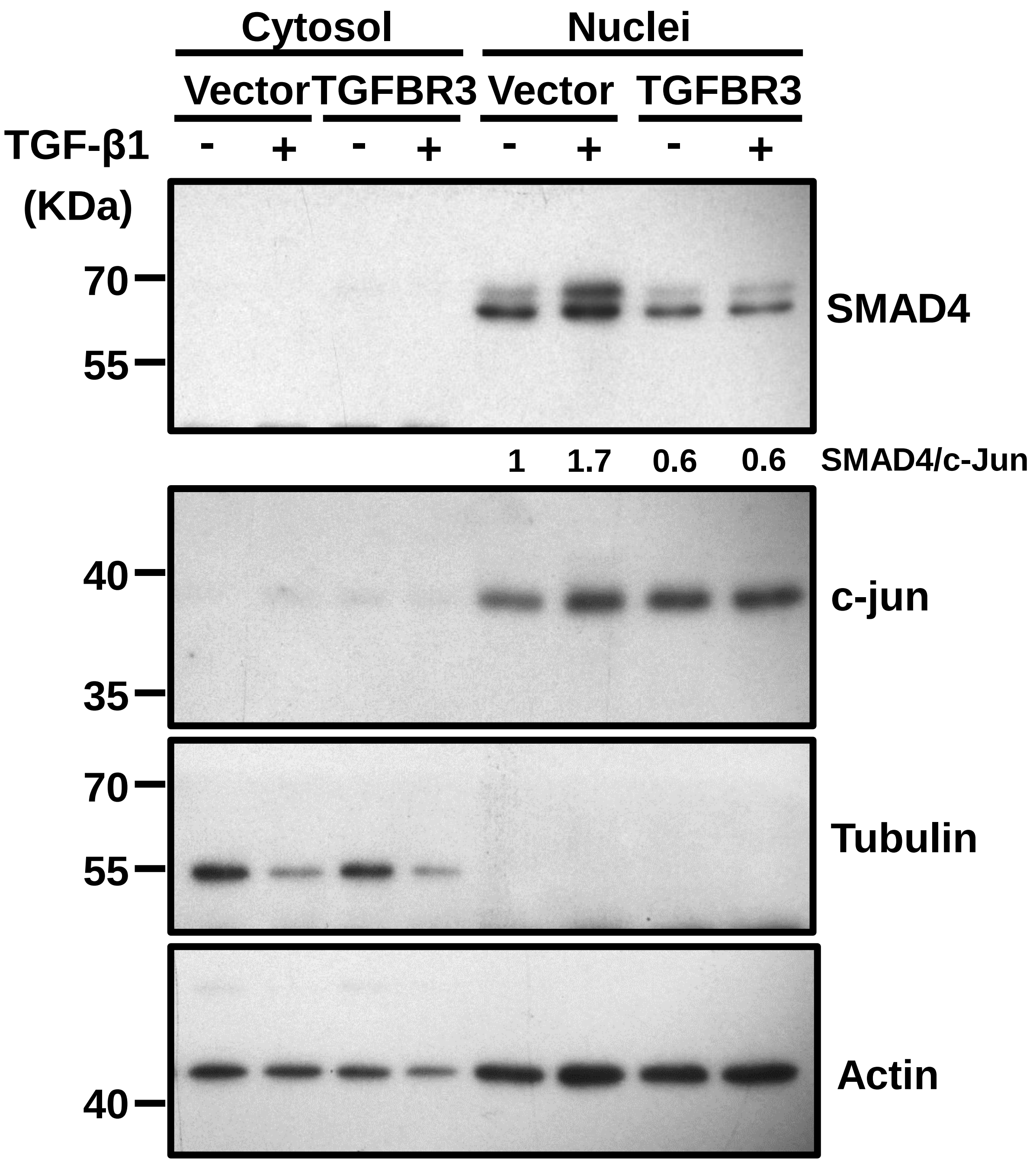

Figure S14 by Fang WY et al

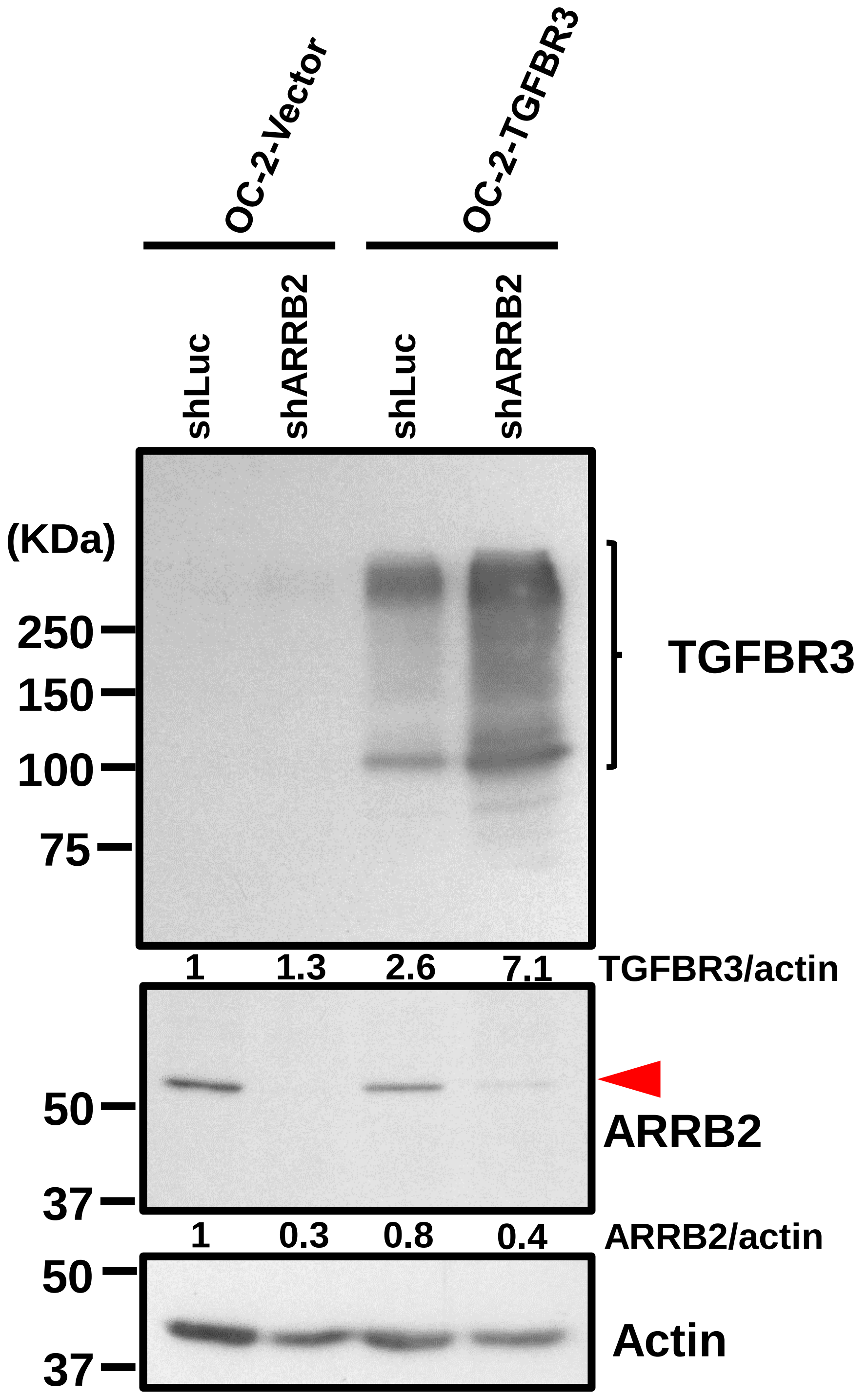

Figure S15 by Fang WY et al

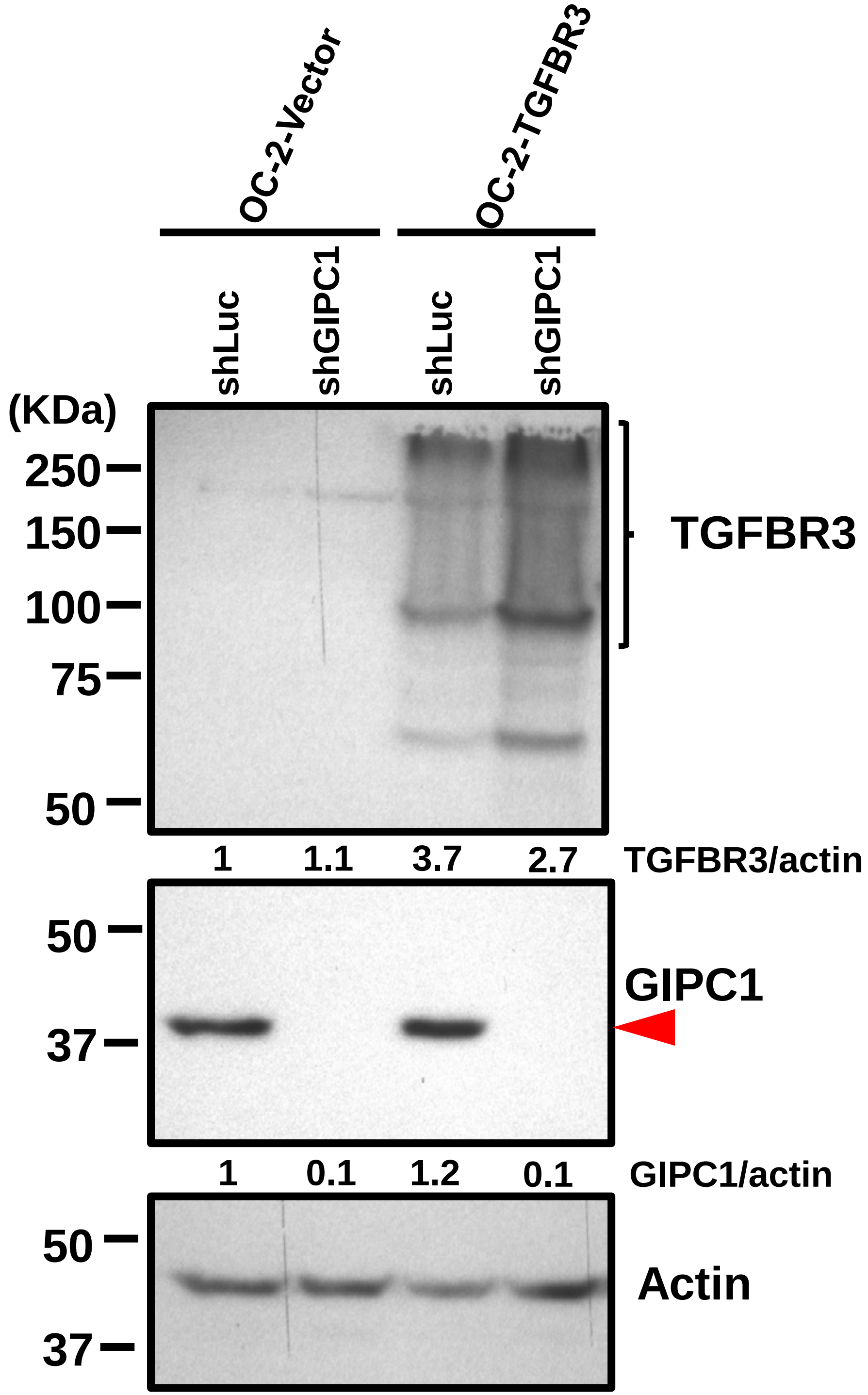

Figure S16 by Fang WY et al

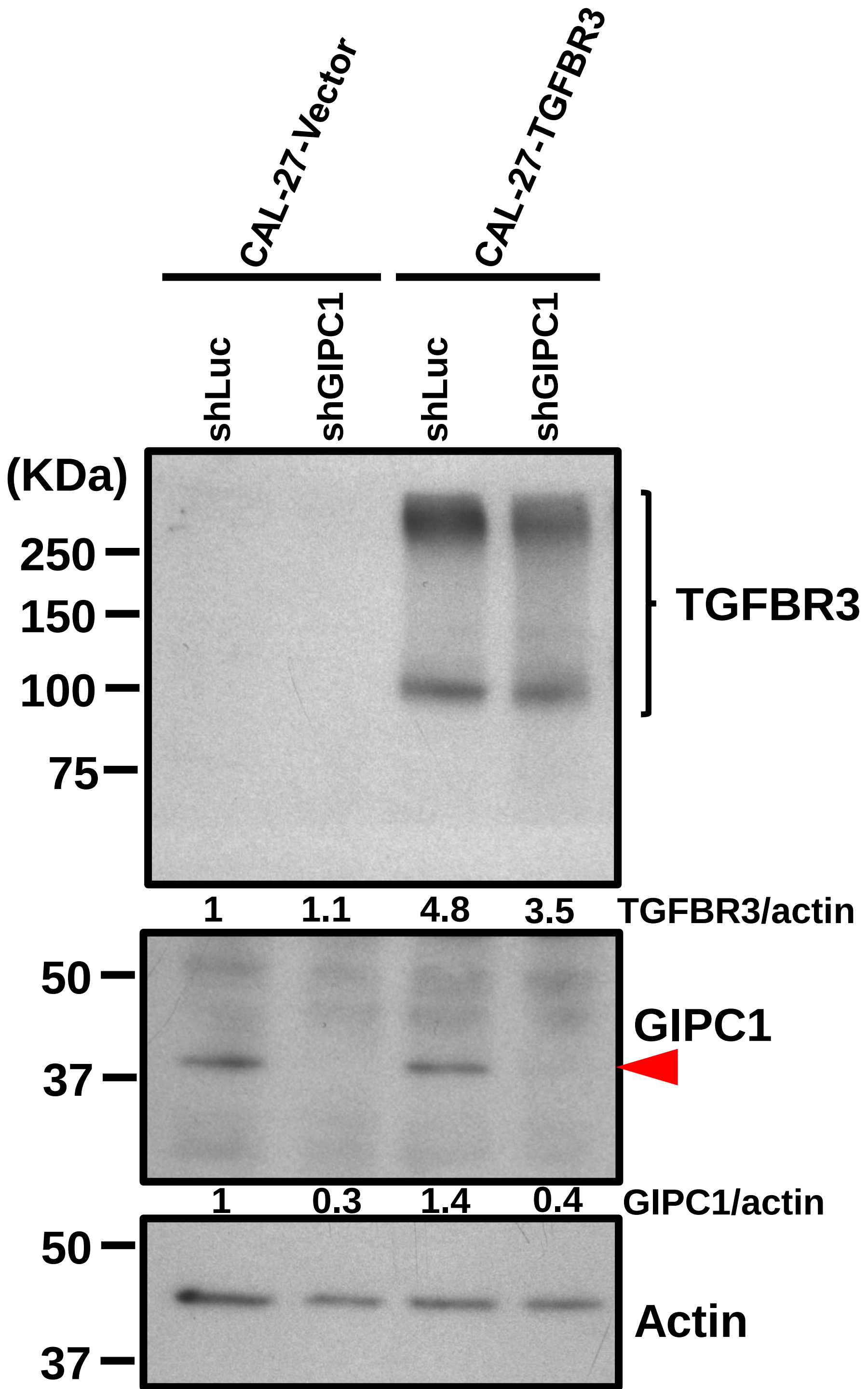

Figure S17 by Fang WY et al

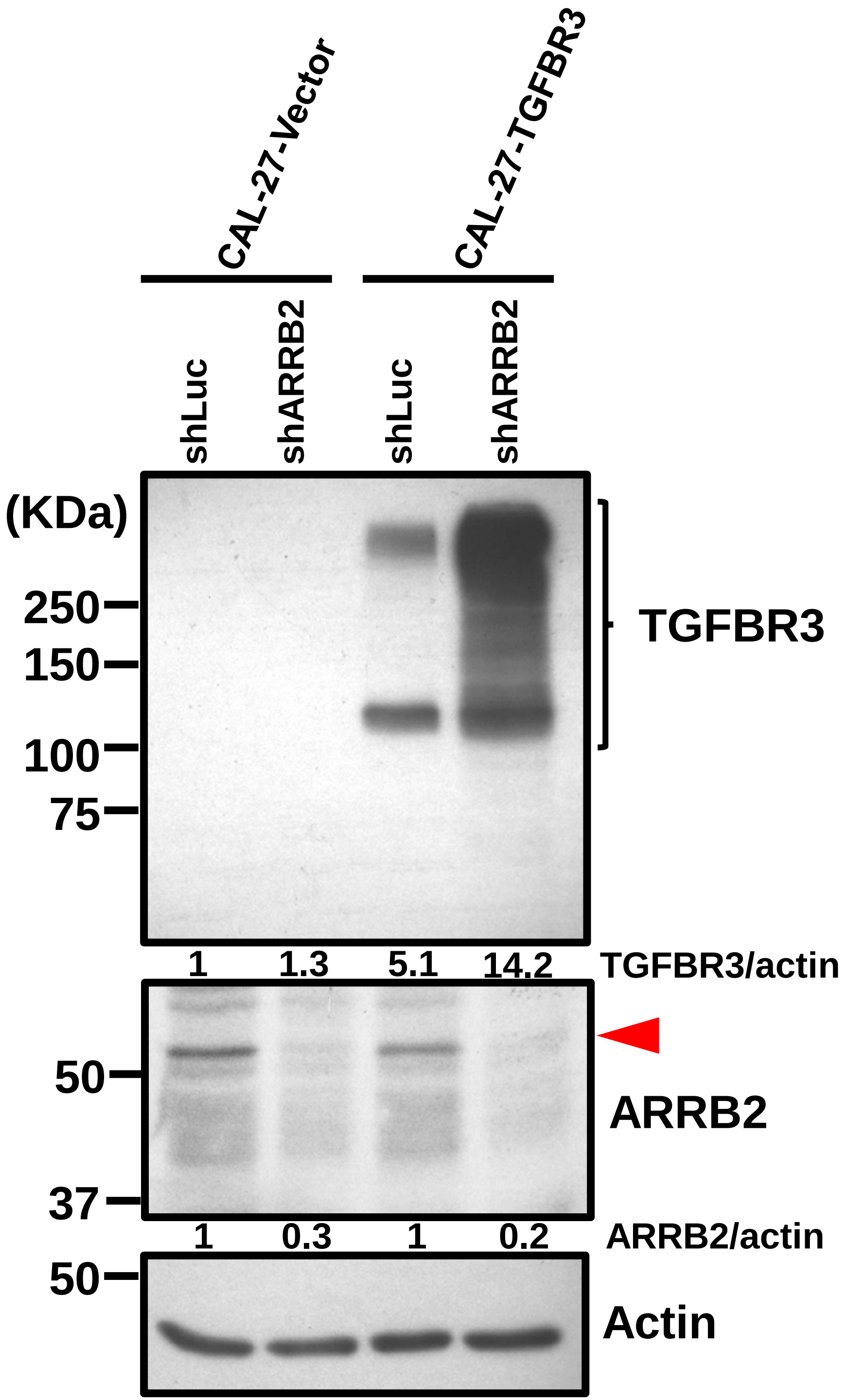

Figure S18 by Fang WY et al

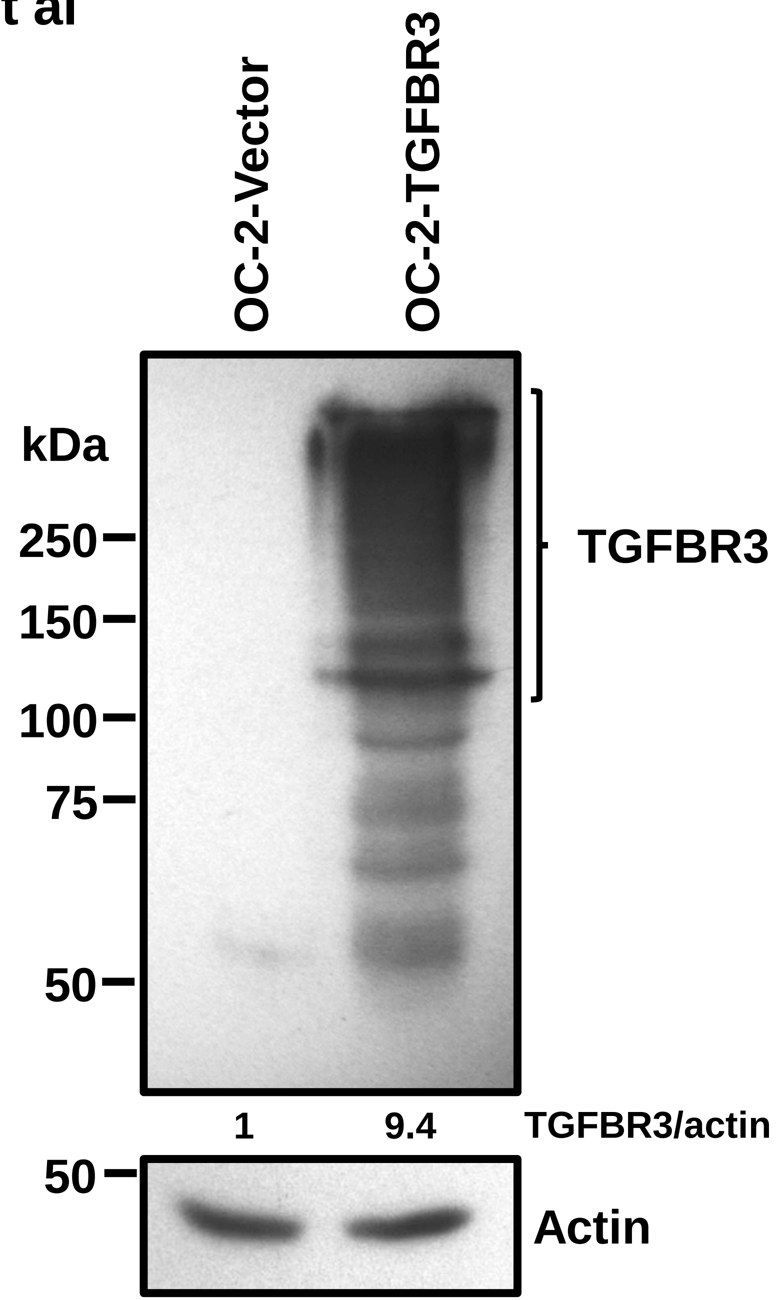

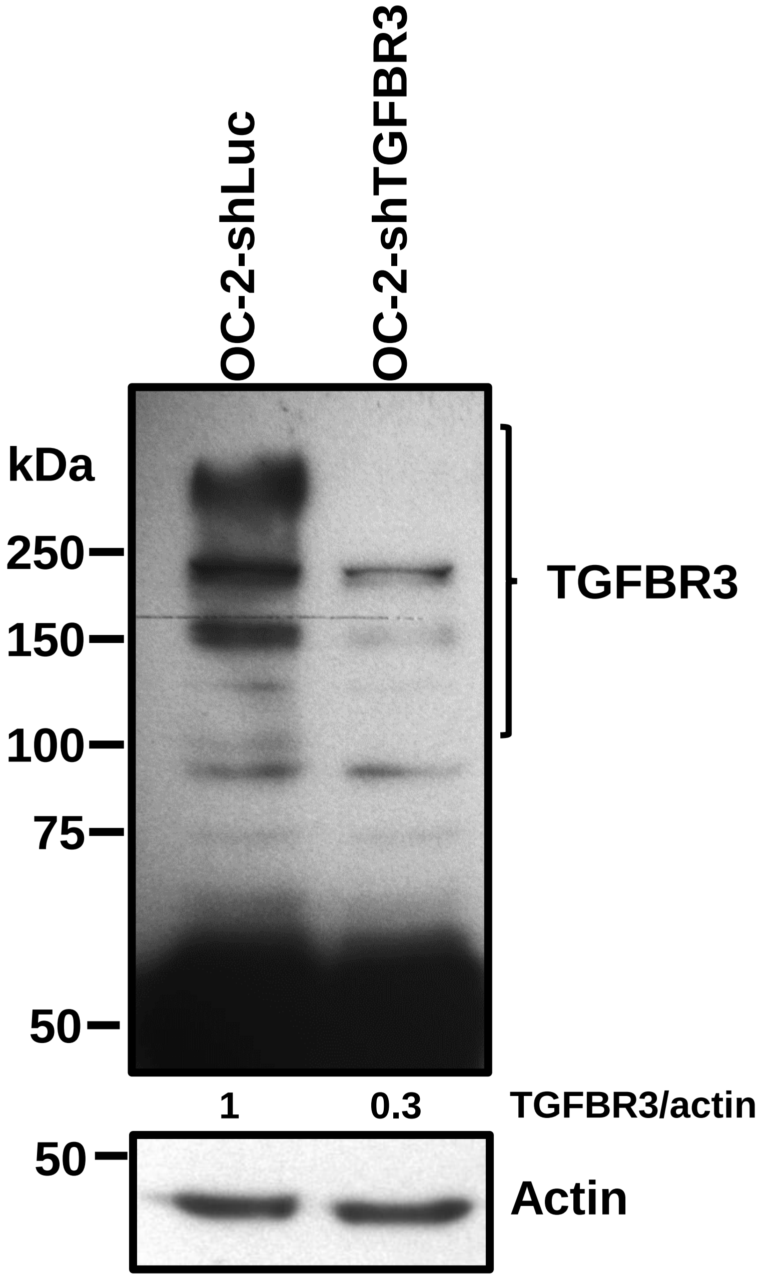

Figure S19 by Fang WY et al

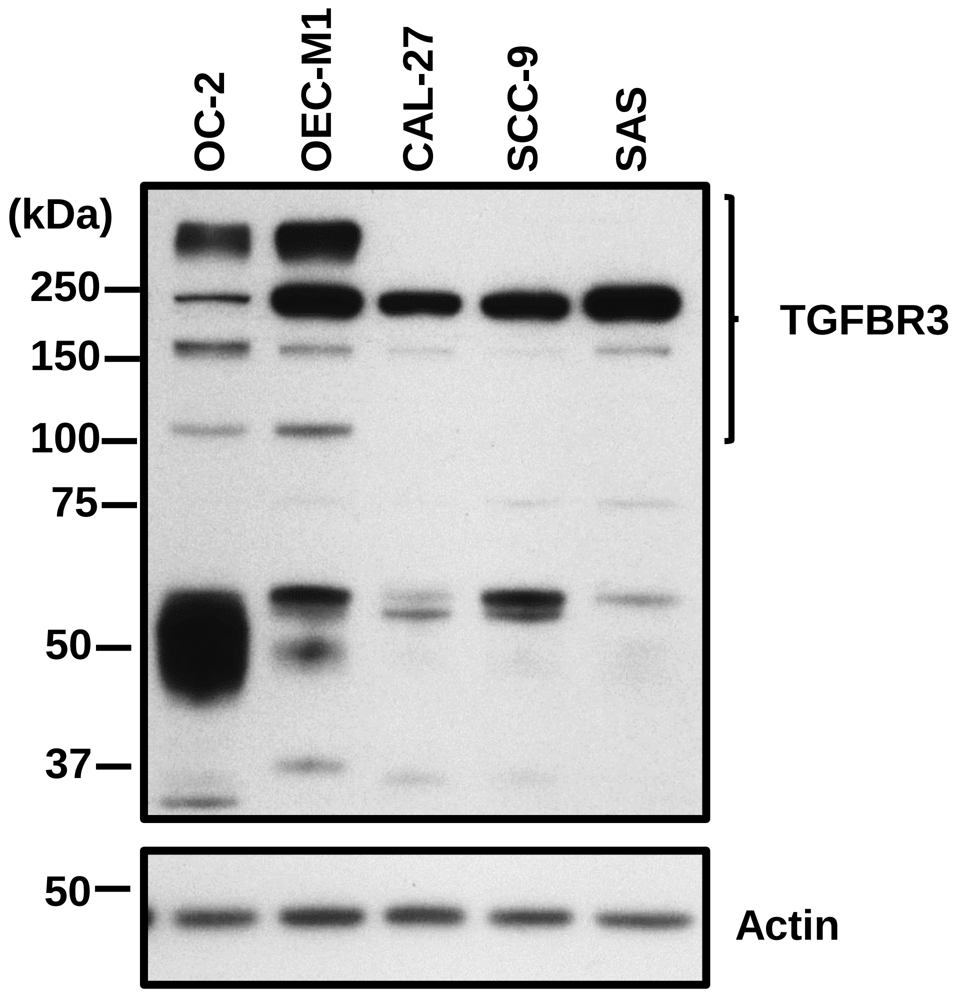

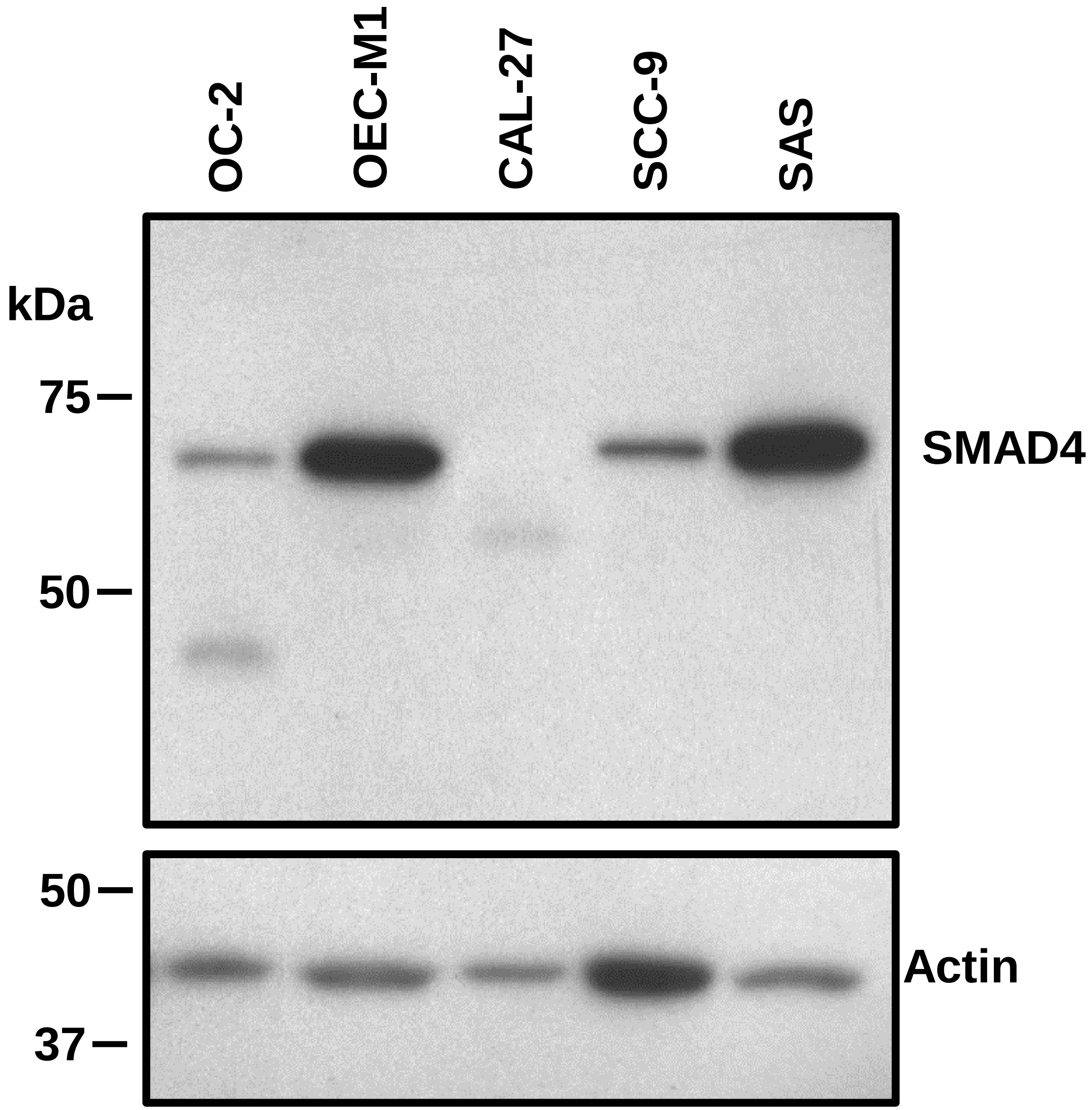

Figure S20 by Fang WY et al

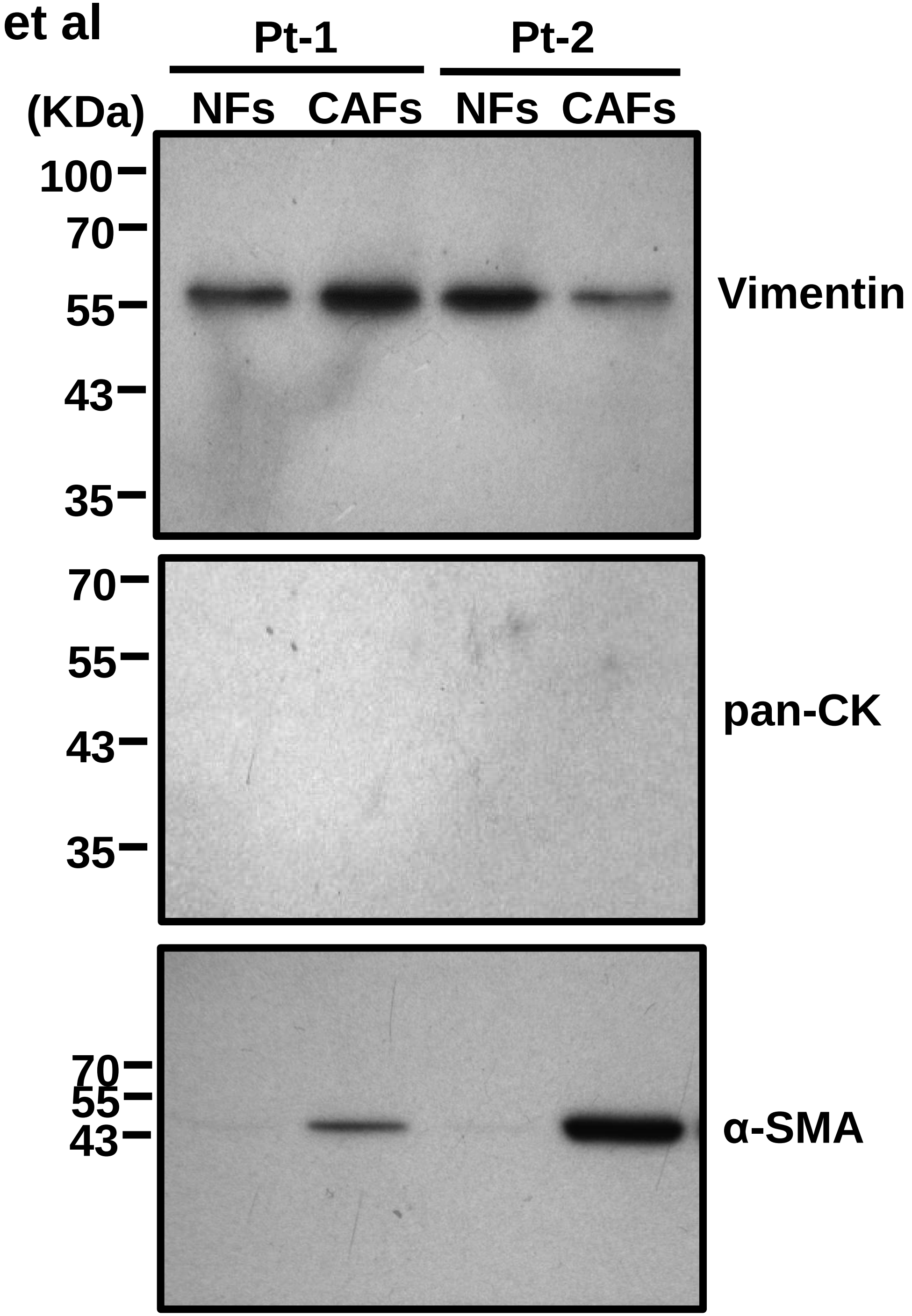

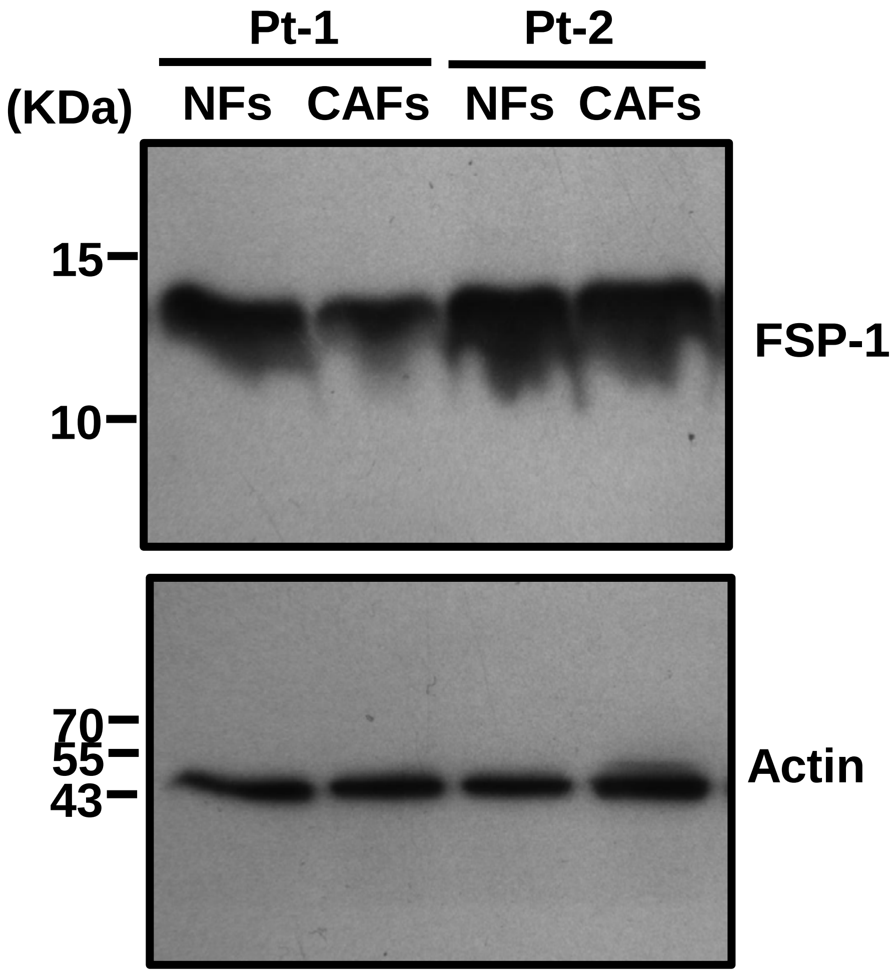

Figure S21 by Fang WY et al

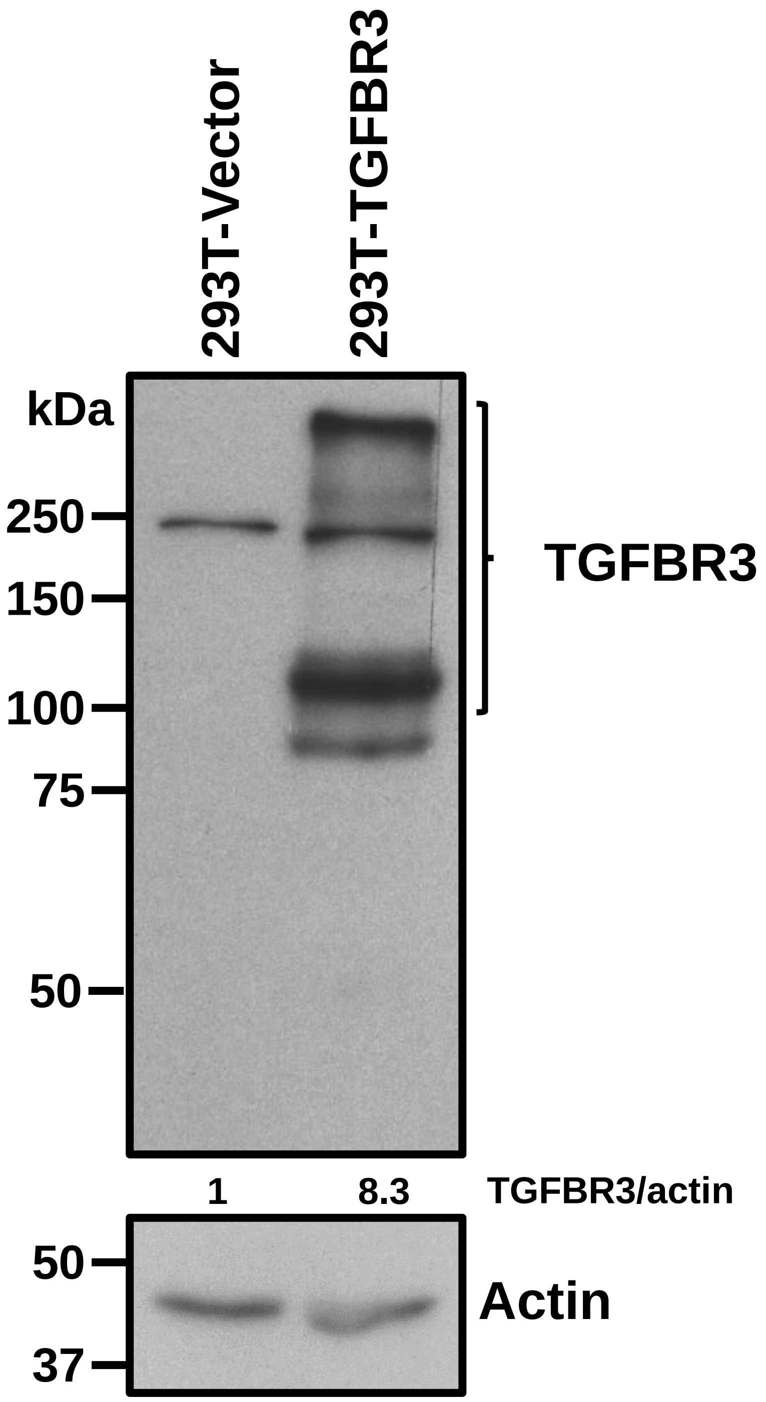

Supplement: Supplementary file 1 [file cancers-12-01375-s001.pdf]
